# The isolated carboxy-terminal domain of human mitochondrial leucyl-tRNA synthetase rescues the pathological phenotype of mitochondrial tRNA mutations in human cells

**DOI:** 10.1002/emmm.201303198

**Published:** 2014-01-10

**Authors:** Elena Perli, Carla Giordano, Annalinda Pisano, Arianna Montanari, Antonio F Campese, Aurelio Reyes, Daniele Ghezzi, Alessia Nasca, Helen A Tuppen, Maurizia Orlandi, Patrizio Di Micco, Elena Poser, Robert W Taylor, Gianni Colotti, Silvia Francisci, Veronica Morea, Laura Frontali, Massimo Zeviani, Giulia d'Amati

**Affiliations:** 1 Department of Radiology, Oncology and Pathology, Sapienza University of RomeRome, Italy; 2 Pasteur Institute-Cenci Bolognetti FoundationRome, Italy; 3 Department of Internal Medicine and Medical Specialties, Sapienza University of RomeRome, Italy; 4 Department of Biology and Biotechnologies ‘Charles Darwin’, Sapienza University of RomeRome, Italy; 5 Department of Molecular Medicine, Sapienza University of RomeRome, Italy; 6 MRC-Mitochondrial Biology UnitCambridge, UK; 7 Unit of Molecular Neurogenetics, The Foundation “Carlo Besta” Institute of Neurology IRCCSMilan, Italy; 8 Wellcome Trust Center for Mitochondrial Research, Institute for Ageing and Health, Newcastle UniversityNewcastle upon Tyne, UK; 9 Department of Biochemical Sciences “A. Rossi Fanelli”, Sapienza University of RomeRome, Italy; 10 National Research Council of Italy, Institute of Molecular Biology and PathologyRome, Italy

**Keywords:** aminoacyl-tRNA synthetases, mitochondrial disease, mitochondrial tRNA mutations, molecular therapy

## Abstract

Mitochondrial (mt) diseases are multisystem disorders due to mutations in nuclear or mtDNA genes. Among the latter, more than 50% are located in transfer RNA (tRNA) genes and are responsible for a wide range of syndromes, for which no effective treatment is available at present. We show that three human mt aminoacyl-tRNA syntethases, namely leucyl-, valyl-, and isoleucyl-tRNA synthetase are able to improve both viability and bioenergetic proficiency of human transmitochondrial cybrid cells carrying pathogenic mutations in the mt-tRNA^Ile^ gene. Importantly, we further demonstrate that the carboxy-terminal domain of human mt leucyl-tRNA synthetase is both necessary and sufficient to improve the pathologic phenotype associated either with these “mild” mutations or with the “severe” m.3243A>G mutation in the mt-tRNA^L^^eu(UUR)^ gene. Furthermore, we provide evidence that this small, non-catalytic domain is able to directly and specifically interact *in vitro* with human mt-tRNA^Leu(UUR)^ with high affinity and stability and, with lower affinity, with mt-tRNA^Ile^. Taken together, our results sustain the hypothesis that the carboxy-terminal domain of human mt leucyl-tRNA synthetase can be used to correct mt dysfunctions caused by mt-tRNA mutations.

## Introduction

Mitochondrial (mt) diseases are multisystem disorders due to mutations in nuclear or mtDNA genes. Among the latter mutations, more than 50% are located in transfer RNA (tRNA) genes (MITOMAP: A Human Mitochondrial Genome Database. http://www.mitomap.org, 2013, and http://www.ncbi.nlm.nih.gov/pubmed/21935892), and are responsible for a wide range of syndromes, such as the severe Mitochondrial Encephalopathy with Lactic Acidosis and Stroke-like episodes (MELAS) for which no effective treatment is available at present.

Experimental evidence has shown that the detrimental effects of mt-tRNA point mutations in human cells can be attenuated by modulating the expression of aminoacyl-tRNA synthetases (aaRSs) (Park *et al*, 2008; Rorbach *et al*, 2008; Li & Guan, 2010; Perli *et al*, 2012). These are ubiquitously expressed, essential enzymes performing the attachment of amino acids to their cognate tRNA molecules, the first step of protein synthesis. Depending on the cellular compartment and the set of tRNAs used as substrates, aaRSs can be specific to cytoplasm, mitochondria, or, in some cases, both. Furthermore, aaRSs are grouped in two classes, based on their structural and functional properties (Ibba & Soll, 2000; Antonellis & Green, 2008; Suzuki *et al*, 2011). Class I aaRSs (specific for aminoacids Val, Leu, Ile, Met, Cys, Glu, Gln, Tyr, Trp and Arg) are mostly monomeric and contain a nucleotide-binding catalytic domain with a Rossman fold, made of six parallel β-strands alternating to α-helices (Li *et al*, 1992; Martinis & Boniecki, 2010). They approach the end of the tRNA acceptor helix from the minor groove side and catalyse the attachment of the aminoacid to the 2′-OH at the 3′-end of the tRNA chain. Class II aaRSs (specific for amino acids Gly, Ala, Ser, Thr, Asn, Asp, Lys, His, Phe and Pro) are mostly dimeric or multimeric and contain an anti-parallel β-sheet flanked by α-helices (Perona *et al*, 1993) sharing at least three conserved regions (Cusack *et al*, 1991; Schimmel, 1991; Delarue & Moras, 1993). Class II aaRS's generally approach the end of the tRNA from the major groove, attaching the aminoacid to the 3′-OH. Class I and Class II are further divided into a, b, c subclasses, each comprising enzymes sharing sequence, structure and function similarities.

A role for mt aminoacyl-tRNA synthetases has been established in human disease (Suzuki *et al*, 2011), since several of them have been reported to carry mutations in patients with a spectrum of different clinical phenotypes (Suzuki *et al*, 2011; Bayat *et al*, 2012; Elo *et al*, 2012; Steenweg *et al*, 2012). Mutations can affect the mt-aaRSs catalytic or editing domain (Scheper *et al*, 2007; Riley *et al*, 2010; Götz *et al*, 2011), the ATP binding site (Elo *et al*, 2012) or the mt targeting sequence (Messmer *et al*, 2011), or may result in immature, truncated proteins (Edvardson *et al*, 2007). Severe reduction in steady-state levels of the corresponding mt-tRNA has been reported as an associated feature (Edvardson *et al*, 2007; Belostotsky *et al*, 2011).

The ability of overexpressed cognate mt-aaRS to attenuate the detrimental effects of mt-tRNA point mutations has been demonstrated both in yeast and human cell lines (De Luca *et al*, 2006, 2009; Park *et al*, 2008; Rorbach *et al*, 2008; Li & Guan, 2010). In particular, studies on human cells (Park *et al*, 2008; Li & Guan, 2010) have shown that overexpression of mt leucyl-tRNA synthetase (mt-LeuRS) corrects the respiratory chain deficiency of transmitochondrial cybrids harbouring the MELAS-associated m.3243A>G mutation in the mt-tRNA^Leu(UUR)^ gene ( *MTTL1*). Likewise, steady-state levels of mutated mt-tRNA^Val^ were partially restored by overexpressing the cognate mt valyl-tRNA synthetase (mt-ValRS) in cybrid cell lines (Rorbach *et al*, 2008). Recently, we showed that constitutive high levels of mt isoleucyl-tRNA synthetase (mt-IleRS) are associated with reduced penetrance of the homoplasmic m.4277T>C mt-tRNA^Ile^ mutation, causing hypertrophic cardiomyopathy. Our *in vivo* findings were paralleled by results in mutant transmitochondrial cybrids following overexpression of mt-IleRS (Perli *et al*, 2012).

In yeast, the overexpression of either human mt-LeuRS or mt-ValRS is capable of rescuing the pathological phenotype associated with human equivalent point mutations in mt-tRNA^Leu^ and mt-tRNA^Val^. In other words, the expression of either enzyme (both belonging to Class Ia) rescued the phenotypes determined by mutations in either the cognate or the non-cognate yeast mt-tRNA. This cross-activity was proposed to reside in regions of mt-LeuRS and mt-ValRS involved in the interactions with tRNA molecules (Montanari *et al*, 2010).

More recently, a region necessary and sufficient to rescue the defective phenotype of human-equivalent mutations in yeast mt-tRNA^Leu^, mt-tRNA^Val^ and mt-tRNA^Ile^ has been identified in the carboxy-terminal domain of yeast mt-LeuRS (Francisci *et al*, 2011).

In the present study, we extended the results obtained in the yeast model to human transmitochondrial cybrid cells (herein named cybrids), by showing the interchangeable ability of three human mt-aaRS, namely mt-ValRS, mt-LeuRS and mt-IleRS, to suppress the mitochondrial functional defects associated with pathogenic homoplasmic mutations in mt-tRNA^Ile^ gene ( *MTTI*). Importantly, we also demonstrate that phenotype rescue can be obtained for either “mild” or “severe” mutations by overexpressing the isolated carboxy-terminal domain of human mt-LeuRS.

## Results

### Pathological phenotype of m.4277T>C and m.4300A>G *MTTI* mutant cybrids

We used previously established osteosarcoma-derived (143B.TK^-^) cybrid cell lines from patients (Perli *et al*, 2012) and controls (Ghelli *et al*, 2009; Pello *et al*, 2008; generous gift from Dr Monica Montopoli and Dr Valerio Carelli) (supplementary Table S1). Both the m.4277T>C and m.4300A>G were previously confirmed as being homoplasmic in all cell lines (Perli *et al*, 2012). To unravel the pathological phenotype, mutant cybrids were grown in glucose-free medium supplemented with galactose (galactose medium), a condition forcing cells to rely on the mt respiratory chain for ATP synthesis. As previously reported, in this condition the selected mutant cybrids clones consistently show a significant reduction in oxygen consumption and viability, the latter due to increased apoptotic death (Perli *et al*, 2012). As expected, cybrids transiently transfected with mock vectors (see Materials and Methods) retained the pathological phenotype (supplementary Fig S1).

### Phenotype correction of *MTTI* cybrids by overexpressing non-cognate human mt-ValRS and mt-LeuRS

**Figure 1:**
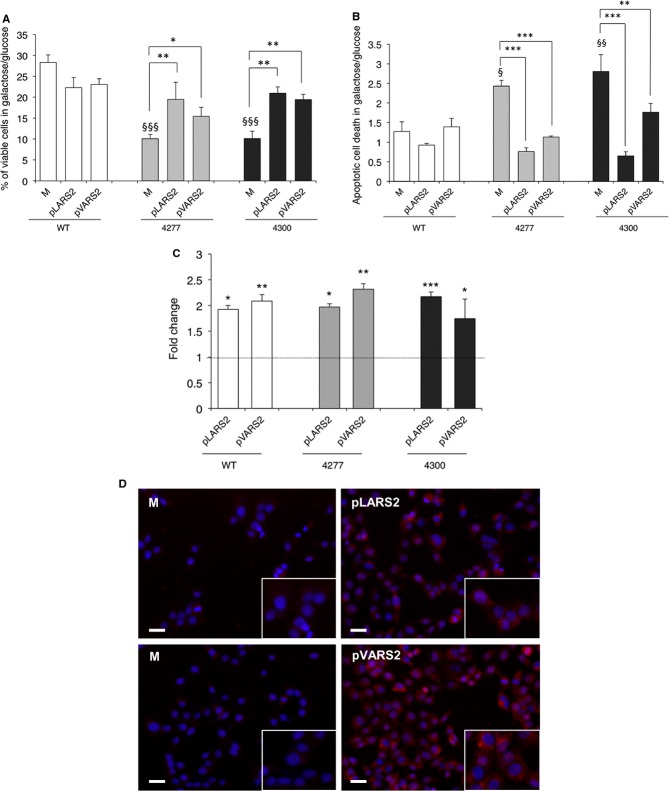
The ability of overexpressed human mt-ValRS and mt-LeuRS to correct mt dysfunction of mutant cybrids carrying the m.4277T>C and m.4300A>G mutations in MTTI gene is interchangeable. Viability of mock, LeuRS and ValRS transformants (M, pLARS2 and pVARS2) evaluated after 48 h incubation in galactose medium. The number of viable cells in galactose is normalized to the number of viable cells in glucose at the same time point. Results are the mean ± s.e.m. of triplicate transfection experiments on *n* = 2 WT, *n* = 2 m.4277T>C and *n* = 2 m.4300A>G cell lines.Apoptotic cell death of M, pLARS2 and pVARS2 evaluated after 24 h incubation in galactose medium and expressed as a ratio between the percentage of apoptotic cells in galactose and in glucose medium. Results are the mean ± s.e.m. of at least duplicate transfection experiments on *n* = 2 WT, *n*=2 m.4277T>C and *n* = 2 m.4300A>G cell lines.Relative expression levels of *LARS2* and *VARS2* genes in trasformants cybrids with respect to *HPRT1* gene. Gene expression levels are normalized to the gene expression level of the respective mock (indicated by the line).Representative images showing expression levels of mt-LeuRS and mt-ValRS in transformant cybrids carrying the m.4277T>C mutation. Top panels: immunofluorescence of M and pLARS2 with a specific anti mt-LeuRS antibody (HPA035951). Bottom panels: M and pVARS2 stained with an anti mt-ValRS specific antibody (15776-1-AP) (see Materials and Methods). Nuclei are stained with DAPI. (Scale bar: 15 μm). Data information: * *P* < 0.05, ** *P* < 0.01, *** *P* < 0.001; ^§^ *P* < 0.05, ^§§^ *P* < 0.001, ^§§§^ *P* < 0.0001 for mutant versus WT mock cells (ANOVA test).

We previously showed that transient overexpression of cognate mt-IleRS determines a 1.5-fold increase in the viability of m.4277T>C *MTTI* mutant cybrids grown in galactose medium (Perli *et al*, 2012). We have obtained similar results with cybrids carrying the homoplasmic m.4300A>G *MTTI* mutation (supplementary Fig S2). We now show that overexpression of non-cognate mt-ValRS and mt-LeuRS ( *VARS2* and *LARS2* genes, respectively) increases the viability of both cybrid lines after 48 h of galactose incubation (up to 2-fold as compared to mock transformants) and decreases apoptotic cell death after 24 h (up to 2.5-fold decrease as compared to mock transformants) (Fig 1A and B).

For each experiment, we measured the transfection efficiency by quantitative real-time PCR (Fig 1C). We found a comparable increase in mRNA expression for mt-LeuRS (mean: +2-fold, range: 1.92–2.17) and mt-ValRS (mean: +2-fold, range: 1.74–2.31) transformants, associated with increased levels of the corresponding proteins, evaluated by immunofluorescence (Fig 1D). Furthermore mt-aaRSs overexpression had no significant effect on viability of wild-type (WT) cybrids (Fig 1A and B).

### The carboxy-terminal domain of human mt-LeuRS is necessary and sufficient to correct the phenotype of *MTTI* mutant cybrids

**Figure 2:**
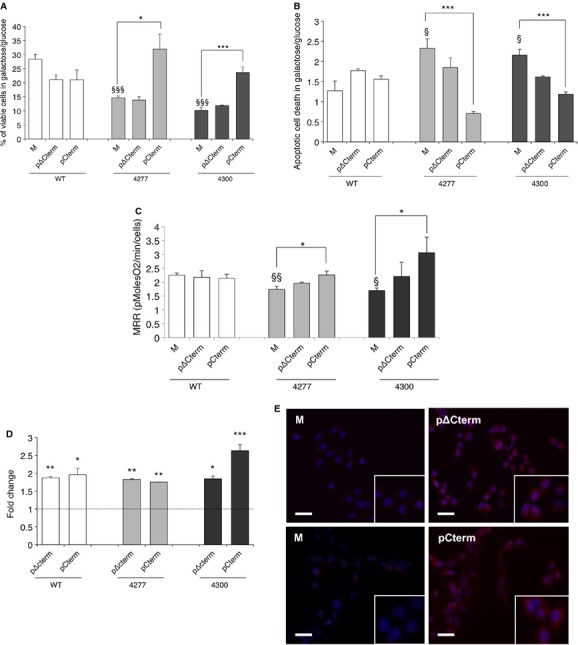
The Cterm domain is both necessary and sufficient to improve cell viability and mitochondrial bioenergetics of cybrids carrying the m.4277T>C and m.4300A>G mutations in MTTI gene. Viability of mock, ΔCterm and Cterm transformants (M, pΔCterm and pCterm) evaluated after 48 h incubation in galactose medium. The number of viable cells in galactose medium is normalized to the number of viable cells in glucose at the same time point. Results are the mean ± s.e.m. of triplicate transfection experiments on *n* = 2 WT, *n* = 2 m.4277T>C and *n* = 2 m.4300A>G cell lines.Apoptotic cell death of M, pΔCterm and pCterm evaluated after 24 h incubation in galactose medium and expressed as ratio between the percentage of apoptotic cells in galactose and in glucose medium. Results are the mean ± s.e.m. of at least duplicate transfection experiments on *n* = 2 WT, *n* = 2 m.4277T>C and *n* = 2 m.4300A>G cell lines.Maximal respiration rate (MRR) measured in M, pΔCterm and pCterm. Values are expressed as ratio of MRR in galactose and in glucose medium. MRR of pCterm is increased by 0.3-fold for m.4277T>C and 0.8 fold for m.4300A>G cybrids as compared to their respective mocks. Results are the mean ± s.e.m. of triplicate transfection experiments on *n* = 1 WT, *n* = 1 m.4277T>C and *n* = 1 m.4300A>G cell lines.Relative expression levels of ΔCterm and Cterm in trasformant cybrids with respect to *HPRT1* gene. Gene expression levels of ΔCterm and Cterm are normalized to the gene expression level of the respective mock (indicated by the line).Representative images showing the expression levels of ΔCterm and Cterm in transformant cybrids carrying the m.4277T>C mutation. Top panels: immunofluorescence of M and pΔCterm stained with a specific anti-mt-LeuRS antibody (HPA035951). Bottom panels: M and pCterm stained with an anti-mt-LeuRS specific for C-terminal region (17097-1-AP) (see Materials and Methods). Nuclei are stained with DAPI. (Scale bar: 15 μm). Data information: * *P* < 0.05, ** *P* < 0.01, *** *P* < 0.001; ^§^ *P* < 0.05, ^§§^ *P* < 0.001, ^§§§^ *P* < 0.0001 for mutant versus WT mock cells (ANOVA test).

Phenotype correction of *MTTI* cybrids by non-cognate mt-aaRS favours the hypothesis that the catalytic function of these enzymes is not primarily involved in the rescuing activity. Based on previous results in yeast, we decided to test the effect of the non-catalytic carboxy-terminal domain of human mt-LeuRS in mutant cybrids. We transiently transfected cybrids with a vector carrying a peptide composed of the 67 C-terminal residues (from amino acid 837 to 903) of human mt-LeuRS (herein named Cterm, see Materials and Methods). Similar to yeast (Francisci *et al*, 2011), galactose-grown transfected mutant cybrids displayed increased viability after 48 h of galactose medium incubation (+2.2 fold relative to mock transformants) and decreased apoptotic cell death after 24 h (up to +2.5) (Fig 2A and B). To investigate whether increased cell viability was indeed related to improved mitochondrial bioenergetics, we measured oxygen consumption rate in transfected cybrids. Using microscale oxygraphy (Invernizzi *et al*, 2012) we clearly demonstrated a significant increase in the maximal respiration rate (MRR) in Cterm transfected mutant cybrids as compared to mock (Fig 2C). Cterm overexpression had no effect on the viability or MRR of WT cybrids.

Finally, overexpression of a human mt-LeuRS variant lacking the C-term domain (ΔCterm) failed to significantly increase cell viability and oxygen consumption rate (Fig 2A and B). Again, we measured transfection efficiency in each experiment (Fig 2D). The mRNA expression increased in both Cterm (mean: 2.1-fold increase, range: 1.74–2.63) and ΔCterm transformants (mean: 1.8-fold increase, range: 1.82–1.86), paralleled by an increase of the corresponding proteins (Fig 2E).

### The carboxy-terminal domain of human mt-LeuRS is able to rescue the phenotype of m.3243A>G MTTL1 mutant cybrids

**Figure 3:**
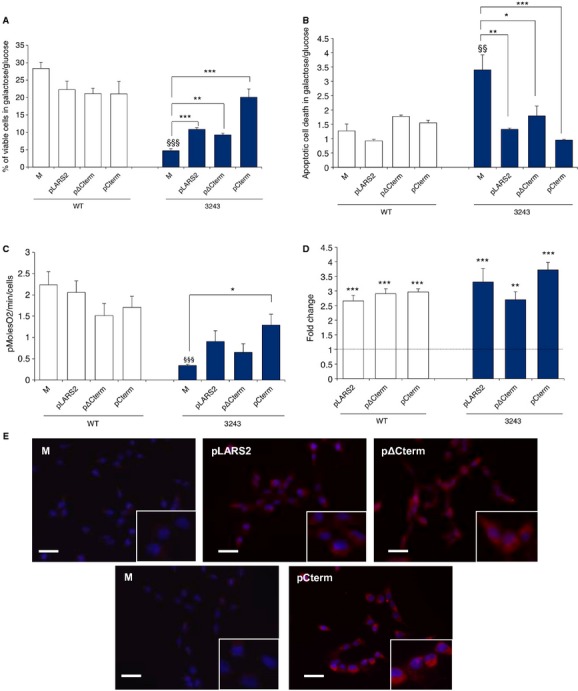
The Cterm domain is able to improve cell viability and mitochondrial bioenergetics of cybrids carrying the severe 3243A>G mutation in MTTL1 gene.. Viability of mock, LARS2, ΔCterm and Cterm transformants (M, pLARS2, pΔCterm and pCterm) evaluated after 48 h incubation in galactose-permissive medium (GPM). The number of viable cells in this condition is normalized to the number of viable cells in glucose at the same time point. Results are the mean ± s.e.m. of triplicate transfection experiments on *n* = 1 WT and *n* = 1 m.3243A>G cell lines.Apoptotic cell death of M, pLARS2, pΔCterm and pCterm evaluated after 24 h incubation in GPM and expressed as ratio between the percentage of apoptotic cells in GPM and in glucose medium. Representative scatter plots are shown in supplementary Fig S4. Results are the mean ± s.e.m. of triplicate transfection experiments on *n* = 1 WT and *n* = 1 m.3243A>G cell lines.Rate of oxygen consumption measured in M, pLARS2, pΔCterm and pCterm maintained in glucose medium. Results are the mean ± s.e.m. of triplicate transfection experiments on *n* = 2 WT and *n* = 1 m.3243A>G cell lines.Relative expression levels of *LARS2*, ΔCterm and Cterm in trasformants cybrids with respect to *HPRT1* gene. Gene expression levels are normalized to the gene expression level of the respective mock (indicated by the line).Representative images showing the expression levels of mt-LeuRS, ΔCterm and Cterm in transformant cybrids carrying the m.3243A>G mutation. Top panels: immunofluorescence of M, pLARS2and pΔCterm stained with a specific anti mt-LeuRS antibody (HPA035951). Bottom panels: M and pCterm stained with an anti mt-LeuRS specific for C-terminal region (17097-1-AP) (see Materials and Methods). Nuclei are stained with DAPI. (Scale bar: 15 μm). Data information: * *P* < 0.05, ** *P* < 0.01, *** *P* < 0.001; ^§§^ *P* < 0.01, ^§§§^ *P* < 0.0001 for mutant versus WT mock cells (ANOVA test).

In order to challenge these results against a severe MTT mutation, we transiently expressed Cterm, ΔCterm and full-length mt-LeuRS protein in cybrid cell lines bearing homoplasmic levels of the m.3243A>G “MELAS” mutation in *MTTL1* (supplementary Table S1). The assessment of m.3243A>G mutation load in cybrids was performed by quantitative pyrosequencing (supplementary Fig S3). As previously demonstrated (King *et al*, 1992) cells carrying high levels of this mutation show severe reduction of the respiratory chain activity and impaired cell growth. In fact, mutant cybrids grown in standard galactose medium undergo massive death within 12 h (see supplementary Fig S4). For this reason we added non-dialyzed FBS to the culture medium (herein galactose permissive medium, GPM) to perform viability experiments. Cybrids transiently overexpressing Cterm displayed increased cell viability after 48 h of incubation in GPM (up to 4 fold as compared to mock transformants) and decreased apoptosis after 24 h (up to 3.5 fold decrease as compared to mock transformants) (Fig 3A, B and supplementary Fig S5). Again, Cterm overexpression did not impact on viability and respiration of WT cybrids.

Interestingly, transfection with the ΔCterm also resulted in a partial phenotype rescuing, suggesting that different regions of mt-LeuRS can contribute to correct the defect associated with the mutated mt-tRNA^Leu(UUR)^. In line with previous observations (Park *et al*, 2008; Li & Guan, 2010) cybrids transfected with the whole enzyme showed significant phenotype improvement (Fig 3A and B).

To assess whether phenotype rescuing reflected an improvement in mitochondrial energetics we evaluated the rate of oxygen consumption of mutant cybrids transfected with the isolated Cterm domain, ΔCterm or whole enzyme as compared to cybrids transfected with the empty vector. Whilst all three transfected lines showed an increase in oxygen consumption rate the improvement was significant only for Cterm transformants (Fig 3C). Transfection efficiency was comparable in the three transformants (Fig 3D and E).

We next investigated whether transfection with the Cterm domain had a direct consequence on the mutated mt-tRNA^Leu(UUR)^ *in vivo*. We assessed the steady-state levels of deacylated mt-tRNA^Leu(UUR)^ by high resolution northern blot analysis in WT and m.3243A>G *MTTL1* mutant cybrids transiently transfected with the whole LeuRS enzyme, the Cterm domain and the MTS-Cterm domain. Values were normalized for 5S rRNA. As expected, the steady-state levels of mutated mt-tRNA^Leu(UUR)^ were reduced by ∼50% relative to WT. Transient overexpression of the three constructs did not result in a detectable increase of the mutated mt-tRNA^Leu(UUR)^ steady-state levels (supplementary Fig S6).

Taken together, these results demonstrate that the Cterm domain can rescue the defective OXPHOS phenotype of MELAS cybrids, being in fact more efficient than the full-length mt-LeuRS protein. Intriguingly, functional rescue is not paralleled by a detectable increase in steady-state levels of the mutated tRNA.

### The Cterm domain of human mt-LeuRS localizes to mitochondria

**Figure 4:**
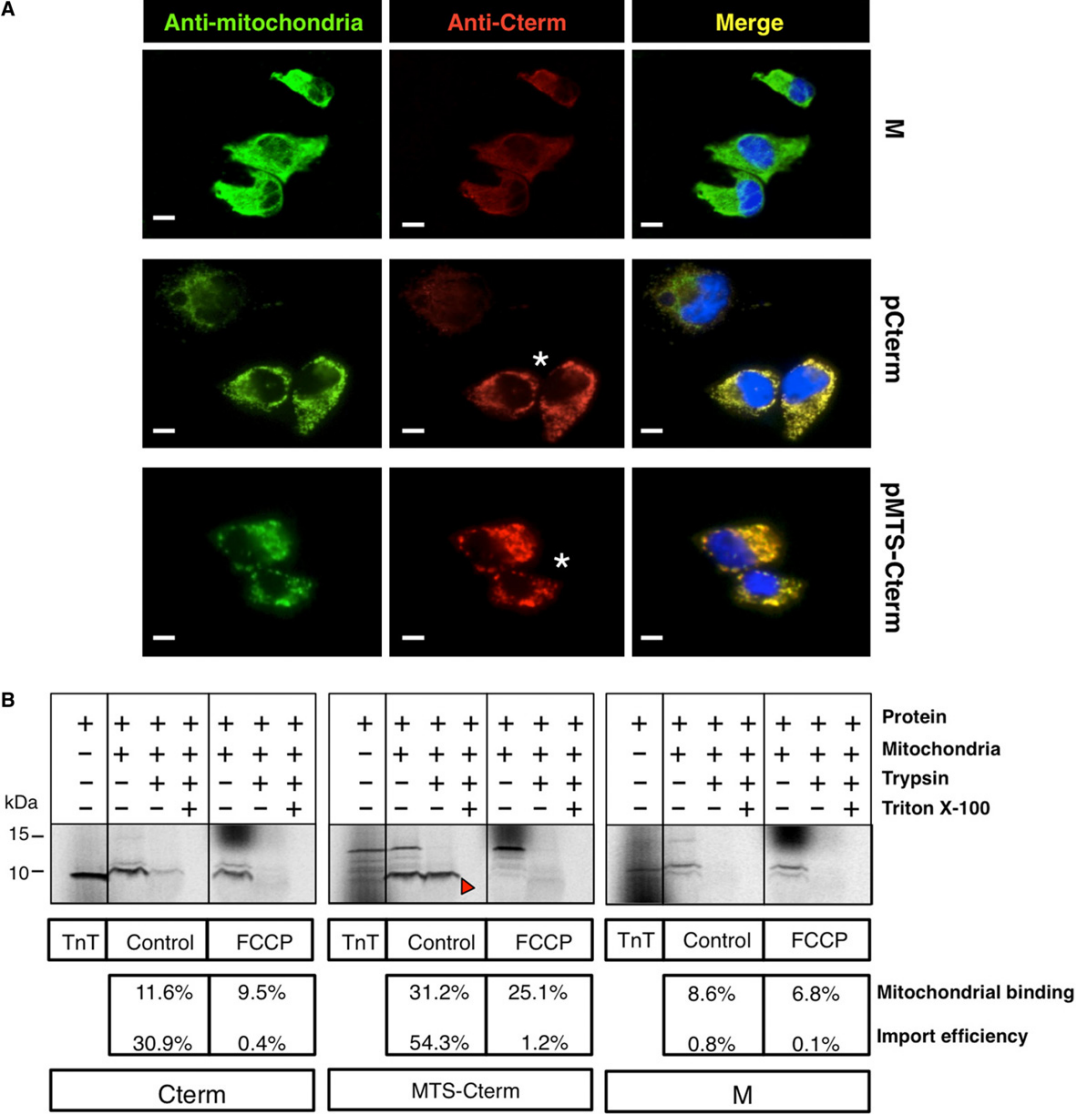
The Cterm domain targets to mitochondria.. Double immunofluorescence stain of mock, Cterm and MTS-Cterm transformants (M, pCterm, pMTS-Cterm) showing colocalization of the antibody against the Cterm region of mt-LeuRS and the antibody against mt extract antigens. Overexpression of the Cterm is highlighted by a stronger mitochondrial immunostaining, as compared to endogenous levels in mock. Highly efficiently transformed cells are marked by an asterisk. Overlap is shown in yellow. Nuclei are stained with DAPI. Cells were examined and imaged with an Olympus IX50 fluorescence microscope. (Scale bar: 15 μm).Import assay of *in vitro* synthesised human peptides Cterm and MTS-Cterm. Incubation of human proteins with isolated rat liver mitochondria in the absence (control) or presence of an uncoupling agent (FCCP) was followed by treatment with either 100  μg/ml trypsin or trypsin plus Triton X-100 and resolved in 18% Tris-glycine SDS–PAGE. The empty vector (M) where the human peptides were cloned was used as a negative control. Same portions of the gels are shown for comparison purposes and TnT reactions are also loaded as a control in each case. The percentage of *in vitro* synthetized peptide able to bind to the mitochondria (mitochondrial binding) and the percentage of imported peptide (import efficiency) are also shown for each assay.

To test whether the rescuing ability of the Cterm is associated with mitochondrial localization, we immunovisualized the peptide both in m.3243A>G transformants and mock, by immunofluorescence with a specific antibody (see Materials and Methods). We also produced a recombinant peptide, composed of the mitochondrial targeting sequence (MTS) of *Neurospora crassa* F0-ATPase subunit 9 precursor (Westermann & Neupert, 2000) fused in frame with the Cterm N-terminus (MTS-Cterm). As shown in Fig 4A, the Cterm-specific immunofluorescence (IF) pattern decorates the mt network and perfectly matches the pattern of MTS-Cterm. Since the antibody used for our IF experiments is specific for a Cterm epitope, we were unable to distinguish endogenous mt-LeuRS from the overexpressed Cterm peptides. However, IF staining intensity in both Cterm and MTS-Cterm transformants was clearly higher than in mock, consistent with overexpression of the peptides.

To corroborate the immunofluorescence findings, we performed mitochondrial import assay of radiolabeled *in vitro*-translated Cterm and MTS-Cterm. As shown in Fig 4B, the Cterm was imported in a membrane potential-dependent manner, with an efficiency of ˜30%. The presence of the MTS improved the efficiency of mitochondrial import up to ˜54%. Following trypsin digestion only the smaller band corresponding to the isolated Cterm was detectable (Fig 4B, arrowhead), consistent with MTS cleavage after translocation into the organelle.

**Figure 5:**
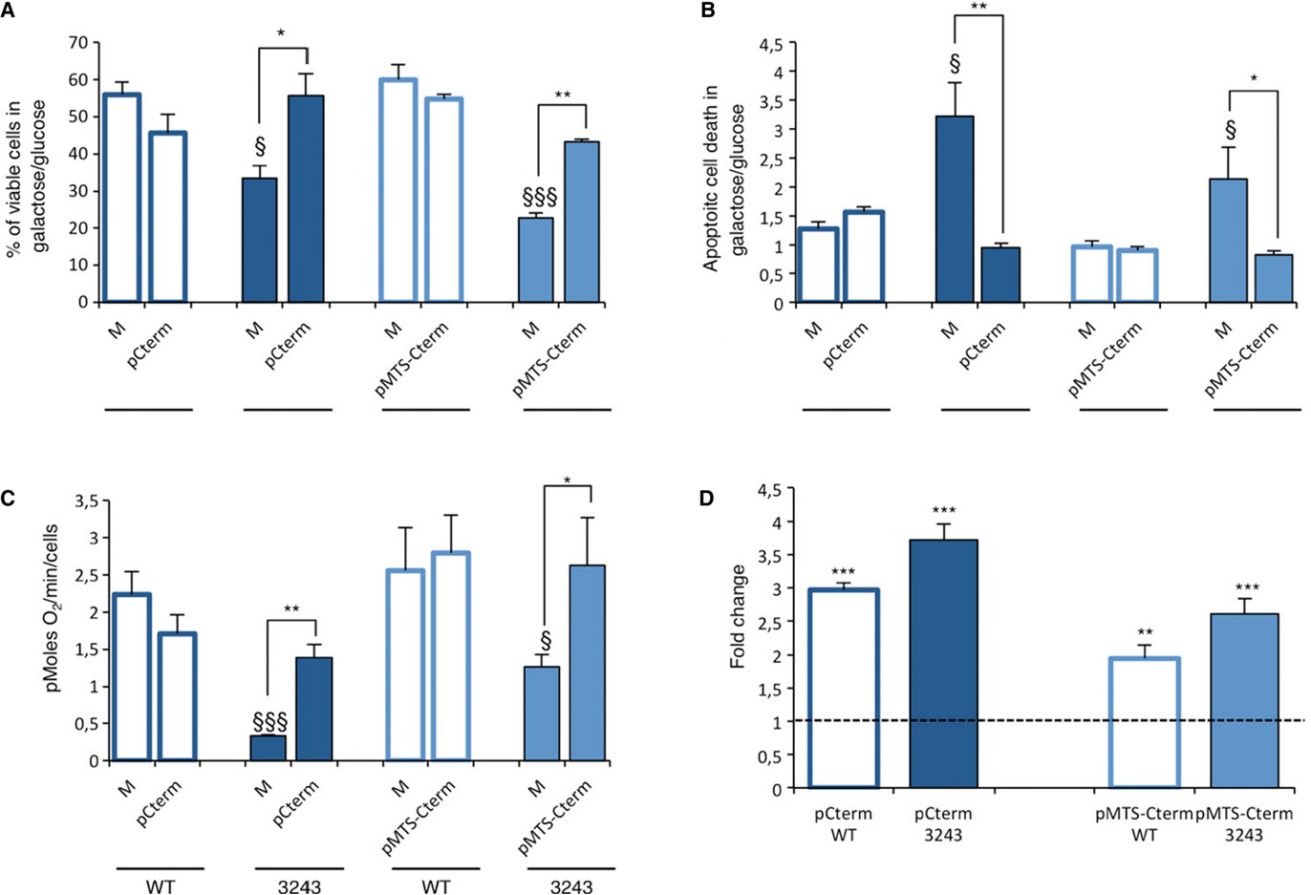
Rescuing effect of Cterm and MTS-Cterm on the m.3243A>G MTTL1 mutant cybrids defective phenotype. Viability of pcDNA5/FRT/TO Cterm (pCterm) and pcDNA3.2 MTS-Cterm (pMTS-Cterm) transformants after 24 h incubation in galactose permissive medium (GPM). The number of viable cells in GPM is normalized to the number of viable cells in glucose at the same time point.Apoptotic cell death after 24 h incubation in GPM and expressed as a ratio between the percentage of apoptotic cells in GPM and in glucose medium.Rate of oxygen consumption of pCterm and pMTS-Cterm transformants maintained in glucose medium. pCterm and pMTS-Cterm transformants are compared with their respective mock (M).Relative expression levels of Cterm and MTS-Cterm with respect to *HPRT1* gene. Gene expression levels are normalized to the gene expression level of the respective M. Data represent mean of three replicates on one mutant and one WT cell line. Data information: * *P* < 0.05, ** *P* < 0.01, *** *P* < 0.001; ^§^ *P* < 0,05, ^§§§^ *P* < 0.0001 for mutant versus WT mock cells (ANOVA test)

Interestingly, the relative increase in mitochondrial import efficiency does not result in higher rescuing activity of the MTS-Cterm. In fact, overexpression of the Cterm — either with or without the MTS — produced comparable effects on viability, apoptotic rate, and oxygen consumption of MELAS cybrids (Fig 5).

The observation that the LeuRS Cterm domain can be imported into mitochondria is not surprising. In fact, for a large fraction of proteins with mt localization a canonical MTS has not been identified (Bolender *et al*, 2008; Li *et al*, 2010). Furthermore, sequence analysis highlighted that both human and yeast C-terminal domains have a favourable positive/negative residues ratio (1.8 and 3.8, respectively), which is higher than in the rest of the protein (0.96 for both human and yeast mt-LeuRS) and similar to that of the N-terminal MTS of the whole enzymes (Zagorski *et al*, 1991; Bullard *et al*, 2000). To investigate the putative spatial location of the positively charged residues we built molecular models of human and yeast Cterm domains. Model inspection showed that, for both domains, positive residues that are distant in the amino acid sequences cluster in the three-dimensional structure to form positively charged patches (supplementary Fig S7).

### The Cterm domain of human mt-LeuRS directly interacts with mt-tRNA^Leu(UUR)^ and mt-tRNA^Ile^ *in vitro*

**Figure 6:**
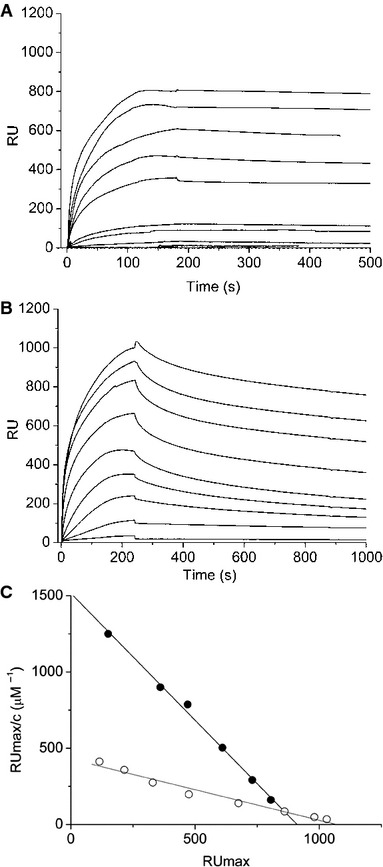
The Cterm peptide directly interacts with human mt-tRNA^Leu (UUR)^ and mt-tRNA^Ile^. Interaction of GST-Cterm with 5′-biotinylated human mt-tRNA^L^^eu(UUR)^ immobilized on a streptavidin-coated sensor chip, measured by Surface Plasmon Resonance experiments. Sensorgrams were obtained using 5′-biotinylated mt-tRNA^L^^eu(UUR)^ as ligand and GST-Cterm (concentration: 10, 20, 40, 80, 120, 400, 600 nM, 1.2, 2.5, 5.0 μM) as analyte.Interaction of GST-Cterm with 5′-biotinylated mt-tRNA^I^^le^ immobilized on a streptavidin-coated sensorchip, measured by Surface Plasmon Resonance experiments. Sensorgrams were obtained using 5′-biotinylated mt-tRNA^I^^le^ as ligand and GST-Cterm (concentration: 150, 300, 600 nM, 1.2, 2.5, 5.0, 10, 20, 30 μM) as analyte.Scatchard plot of the sensorgrams. The resulting K_D_ is 0.6 μM for 5′-biotinylated mt-tRNA^L^^eu(UUR)^ (
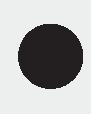
) and 2.5 μM for 5′-biotinylated mt-tRNA^I^^le^ (○).

We tested the ability of Cterm to interact directly with human mt-tRNA^Leu(UUR)^ and mt-tRNA^Ile^ by Surface Plasmon Resonance (SPR). The 5′-biotinylated mt-tRNA^Leu(UUR)^ and 5′-biotinylated mt-tRNA^Ile^ were immobilized on the streptavidin-containing surface of two different SPR sensorchips, and allowed to interact with different concentrations of a purified recombinant construct comprising glutathione S-transferase (GST) protein fused in frame with the Cterm domain (GST-Cterm) (Fig 6A). We found that the affinity of GST-Cterm interaction for 5′-biotinylated mt-tRNA^Leu(UUR)^ is high, the K_D_ of the complex being about 0.6 μM (Fig 6C). In particular, the dissociation rate is very low in our experimental conditions, indicating high stability of the complex. The same experiments were performed with GST alone, at the same concentrations used for GST-Cterm in the experiment in Fig 6A, as a negative control. As expected, GST binding to tRNA was negligible (about 20%, supplementary Fig S8). The affinity of GST-Cterm for 5′-biotinylated mt-tRNA^Ile^ is lower than for 5′-biotinylated mt-tRNA^Leu(UUR)^, the K_D_ of the complex being about 2.5 μM (Fig 6B and C). The same experiments, performed with GST alone, showed that GST binding to tRNA^Ile^ was negligible (about 15%, supplementary Fig S8).

## Discussion

In the present study we show that three human mt-aaRS, namely mt-LeuRS, mt-ValRS, and mt-IleRS, are able to ameliorate the mt defects associated with two mutations in mt-tRNA^Ile^ ( *MTTI*). Importantly, we show that phenotype rescuing is obtained both for these “mild” mutations and for the “severe” m.3243A>G mutation in *MTTL1* gene, by overexpressing the carboxy-terminal domain of human mt-LeuRS (Cterm). The m.4277T>C and m.4300A>G *MTTI* gene mutations cause isolated cardiomyopathy and are found at homoplasmic levels (Taylor *et al*, 2003; Perli *et al*, 2012), whereas the m.3243A>G *MTTL1* gene mutation is heteroplasmic, and can be associated with severe biochemical and clinical phenotypes, such as those ascribed to MELAS (Kaufmann *et al*, 2011) at high mutation loads. Besides the typical MELAS phenotype, the m.3243A>G mutation has been associated with a wide spectrum of clinical manifestations, including maternally-inherited diabetes and deafness (MIDD), partly dictated by the level of m.3243A>G heteroplasmy, and its frequency in some populations is higher than previously thought (Manwaring *et al*, 2007).

Remarkably, overexpression of Cterm was sufficient to improve both viability and bioenergetic proficiency of cybrids bearing mutations in either cognate or non-cognate mt-tRNAs. Moreover, the Cterm had a higher rescuing activity than the whole enzyme towards all of the tested mutations. Conversely, the ΔCterm mt-LeuRS variant had negligible or no effect on the phenotype of cybrids bearing mutations in cognate and non-cognate mt-tRNAs, respectively.

Consistent with the observed functional effect of the Cterm on mutant cybrids, we show that this short (67 residues long) domain is imported into mitochondria. In the absence of a typical N-terminal MTS, the mt localization of overexpressed human Cterm demonstrated in this work may be due to the predominance of positive over negative amino-acid residues in the sequence (Bullard *et al*, 2000; Neupert & Hermann, 2007), and to the spatial clustering of positive residues, as suggested by the three-dimensional homology model of the Cterm.

Interestingly, adding a canonical MTS does not improve the rescuing by the Cterm, despite the observed enhancement in mitochondrial import efficiency.

Taken together, our results sustain the hypothesis that human mt-LeuRS Cterm can be used to correct mt dysfunctions caused by mutations in mt-tRNA genes. Our findings extend to human the results previously obtained in the yeast *Saccharomyces cerevisiae* (De Luca *et al*, 2009; Montanari *et al*, 2010; Francisci *et al*, 2011).

Since our results are relevant for potential therapeutic applications, an investigation of the mechanisms underlying the rescuing activity of the Cterm is called for. In the three-dimensional structures of *Thermus thermophilus* (Tukalo *et al*, 2005) and *Escherichia coli* (Palencia *et al*, 2012) LeuRS–tRNA^Leu^ complexes determined by X-ray crystallography (Protein Data Bank (PDB) IDs: 2BTE and 4AS1, respectively), the Cterm domain of LeuRS interacts with the elbow of the L-shaped tRNA, stabilizing the complex (Hsu & Martinis, 2008; Hu *et al*, 2013). Based on these observations, we previously proposed that the Cterm of yeast LeuRS ( *NAM2* gene) may act as a chaperone (Francisci *et al*, 2011), by directly interacting with the mutant mt-tRNA^Leu^ and stabilizing a conformation suitable to react with the mt-LeuRS and/or less amenable to degradation. Accordingly, we have shown here that Cterm is able to directly and specifically interact with human mt-tRNA^Leu(UUR)^ *in vitro*, with high affinity and stability. Consistent with its rescuing activity in cybrids bearing the m.4300A>G and m.4277T>C mutations, the Cterm interacted *in vitro* with the non-cognate human mt-tRNA^Ile^, although with lower affinity. These results strongly suggest that functional rescuing of mt-tRNA^Leu(UUR)^ and mt-tRNA^Ile^ defects is mediated by physical interactions of LeuRS Cterm with mutant tRNAs.

At variance with previous reports (Park *et al*, 2008; Rorbach *et al*, 2008; Li & Guan, 2010) and the accompanying paper, we did not detect an increase of mutant mt-tRNA^Leu(UUR)^ steady-state levels upon transient overexpression of either Cterm or the whole LeuRS enzyme, despite a significant increase in cell viability and mitochondrial respiration. This finding suggests that the effect of the Cterm is mainly to improve the function of the mutant mt-tRNA^Leu(UUR)^ rather than preventing it from degradation. As a consequence, mt translation and OXPHOS would improve, and apoptosis would be prevented. It is also possible that on a longer timescale, this effect may lead to an increase of the steady-state levels of mt-tRNA, as reported elsewhere, that was not observed in the transient, “acute” experiment shown here.

We also demonstrate that the catalytic activity of human mt-LeuRS is dispensable for the rescuing effect, since the latter is carried out by the short, catalytically incompetent, Cterm domain, in agreement with previously obtained data in yeast model (De Luca *et al*, 2009; Francisci *et al*, 2011).

We propose that the Cterm acts as a chaperone by direct physical contact with the mutated mt-tRNA *in vivo*, helping it to maintain the conformation suitable to proficiently interact with its cognate aminoacyl-tRNA synthetase or other molecular partners.

This hypothesis is in agreement with the higher rescuing activity displayed by the Cterm relative to the whole enzyme, since the reduced steric hindrance of the former is likely to reduce the interference of the tRNA interactions with (a) the cognate aaRS, or (b) other molecular partners, for example the ribosome. For instance, *in silico* analysis of *T. thermophilus* tRNA^Leu^-LeuRS complex, and tRNA^Phe^-mt ribosome complex suggest that only free tRNAs can efficiently get access to the ribosome (Selmer *et al*, 2006; PDB ID: 2J00).

The ability of the short Cterm to rescue both mild and severe defects associated with cognate and non-cognate mt-tRNA mutations opens new perspectives for treatment of human diseases associated with mt-tRNA mutations. For instance, adeno-associated virus-mediated targeting could be exploited to express the isolated Cterm *in vivo*. The efficacy and safety of this approach have been demonstrated both in human (Nathwani *et al*, 2011) and in animal models of genetic disease (Di Meo *et al*, 2012).

Alternatively, the recent demonstration of the rescuing activity obtained by overexpressing the short β30–31 sequence of the human Cterm in the yeast model (Francisci *et al*, 2011) suggests that Cterm-derived peptides may be used as therapeutic agents, provided that suitable agents for mitochondria targeting are employed to deliver them to their subcellular destination. Cterm peptides may even prompt the development of non-peptide organic molecules, especially if the rescuing activity can be further restricted to smaller regions.

In light of a broader application of this therapeutic approach, further experiments are advised to test the effect of mt-LeuRS Cterm and/or smaller peptides thereof on further mutations in mt-tRNAs aminoacylated by class I or II aaRS. In parallel, it will be worth investigating the structural determinants of the cross-rescuing activity of IleRS and ValRS and whether additional aaRSs are endowed with the same ability. In this framework, it should be noted that in the yeast model, while overexpression of whole mt-IleRS rescues the defects due to mutations in both mt tRNA^Ile^ and tRNA^Leu^, the carboxy-terminal domain of IleRS — contrary to the Cterm of mt-LeuRS — does not rescue either mutant (supplementary Fig S9). Accordingly, sequence and structure analyses indicate that there is no obvious homology between the carboxy-terminal domains of LeuRS and IleRS (supplementary Fig S10 A, B and C), suggesting that the determinants of the cross-rescuing activity of different aaRSs are likely to reside in different enzyme regions.

## Materials and Methods

### Cell culture

Cybrids were cultured in Dulbecco's modified Eagle's medium (DMEM), supplemented with 4.5 g/l D-glucose, 10% fetal bovine serum (FBS), 2 mM L-glutamine, 100 U/ml penicillin and 100 mg/ml streptomycin (referred to as glucose medium) in a humidified atmosphere of 95% air and 5% CO_2_ at 37°C. For a subset of experiments cybrids were growth in glucose-free DMEM, supplemented with 5 mM galactose, 110 mg/ml sodium pyruvate and 10% dialyzed FBS (dFBS) (referred to as galactose medium). For a subset of experiments m.3243A>G cybrids were grown in glucose-free DMEM, supplemented with 5 mM galactose, 110 mg/ml sodium pyruvate and 10% FBS (referred to as permissive galactose medium).

### Assessment of m.3243A>G mutation load

The assessment of m.3243A>G mutation load was performed by quantitative pyrosequencing. Pyromark Assay Design Software v.2.0 (Qiagen, Hamburg, Germany) was used to design locus-specific PCR and pyrosequencing primers. A 211 bp PCR product spanning the m.3243 nucleotide was amplified using a biotinylated forward primer (nt 3143–3163) and a reverse primer (nt 3353–3331). Pyrosequencing was carried out on the Pyromark Q24 platform according to the manufacturer's instructions, using a mutation-specific reverse primer (nt 3258–3244). Levels of m.3243A>G heteroplasmy were determined using Pyromark Q24 software, which directly compares the relevant peak heights of both WT and mutant mtDNA at this site.

### Plasmid construction

Standard protocols were used for *E. coli* transformation as well as plasmid preparations (Sambrook *et al*, 1989). The pcDNA5/FRT/TO VARS2 plasmid (pVARS2) was kindly provided by Prof. R.N. Lightowlers (Rorbach *et al*, 2008). The human *LARS2* gene cDNA (consensus CDS 2728.1) was subcloned from pYES2.1/V5-His-TOPO (Montanari *et al*, 2010) into BamHI/XhoI restriction sites of the pcDNA5/FRT/TO plasmid (Invitrogen™, Life Technologies Italia, MB, Italy) (pLARS2). The *LARS2* cDNA deleted of the entire Cterm domain (from residue 837 to 903) was amplified and cloned into the BamHI/XhoI restriction sites of the pcDNA5/FRT/TO (pΔCterm). Both constructs were kindly provided by Prof. R.N. Lightowlers. To produce the pcDNA5/FRT/TO Cterm plasmid (pCterm), the fragment of the *LARS2* cDNA from 2509 to 2712 bp was amplified from pLARS2 using BamHICtermLARS2+ (5′-CGGGATCCCG**ATG**GAGGTTGTCCAGATGGCAGTTC-3′), that contains the additional ATG codon (in bold) and BamHICtermLARS2-(5′-CGGGATCCCG**TCA**ATCTTGCACCAGGAAGTTG-3′) that contains the STOP codon (in bold). In each primer the BamHI restriction site sequence is underlined. The PCR product was cloned into the BamHI restriction site of the plasmid. The final cloned construct contains the initiator Met codon and the 67 terminal amino acids (from residue 837 to 903) of human mt LeuRS. The Cterm domain from human *LARS2* gene, fused at 5′ with the residues 1–69 of *Neurospora crassa* Fo-ATPase subunit 9 precursor (NCBI accession number: XP_959894) and at 3′ with GFP (spaced by a 9 residues linker: LEMESDESG) was cloned into pTUNE plasmid (LifeTechnologies, Carlsbad, CA, USA). Using the latter plasmid as template and the primers listed below, amplicon containing MTS-Cterm and Cterm were obtained and subcloned into pcDNA3.2-TOPO plasmid (Invitrogen™, Life Technologies, Italia, MB, Italy) (pMTS-Cterm and p3.2Cterm).

MTS pcDNA3.2 Fw: 5′-CACCATGGCGAGTACTCGGG-3′; Cterm pcDNA3.2 Fw: 5′-CACCATGGAGGTTGTCCAGATGGCA-3′; Cterm Rc: 5′-TCATCAATCTTGCACCAGGA-3′.

*IARS2* was cloned in pTUNE vector as described in Perli *et al* (2012) (pIARS2).

### Transient transfections

Cybrids were plated in 60-mm dishes, incubated in glucose medium for 24 h, and then transfected with 2 μg of the full or empty vector by using Turbofectin 8 (Origene Technologies, Inc., Rockville, MD, USA) according to the manufacturer's protocol.

Six hours after transfection, 2 mM tetracycline (Sigma, St Louis, MO, USA) was added to the medium of pcDNA5/FRT/TO transformants while 150 μM isopropyl-b-D-1-thiogalactopyranoside (IPTG, Sigma) was added to the medium of pTUNE transformants for vector induction. All the media used for the successive experiments were supplemented with constant concentrations of these inducers.

### Evaluation of transient transfection efficiency by quantitative real-time PCR

About 48 h after transfection total RNA was isolated from transfected cells using SV Total RNA isolation kit (Promega, Fitchburg, WI, USA), measured with a NanoDrop ND-1000 spectrophotometer (NanoDrop Technologies, Wilmington, DE, USA), and then 0.1–1 mg were reverse-transcribed to cDNA using random hexamer primers. To assess the relative expression of *IARS2, VARS2, LARS2* and Cterm, we used TaqMan probe chemistry by means of inventoried and custom FAM-labeled TaqMan MGB probes (Applied Biosystems Warrington, UK; supplementary Table S2), according to the manufacturer's instructions. In all samples, the relative expression of each target gene was evaluated with respect to average of controls (mock sample) using the comparative threshold cycle (Δ *C*_t_) method. All values were normalized for the housekeeper hypoxanthine phosphoribosyltransferase 1 ( *HPRT1*).

### Immunofluorescence and co-localization experiments

About 48 h after transfection cells were plated on cover slips, fixed with 4% formaldehyde freshly prepared from paraformaldehyde in PBS (pH 7.4) with 0.1% Triton X-100. The following primary antibodies were used: rabbit polyclonal antibody anti-mt-LeuRS (HPA035951, ATLAS antibodies, AlbaNova University Center, Stockholm, Sweden) specific for the region spanning the mt-LeuRS catalytic core and anticodon binding domains (NCBI reference sequence NP_056155.1); rabbit polyclonal antibody specific for the Cterm domain of human mt-LeuRS (antigen peptide region 554-903, 17097-1-AP, ProteinTech group, Chicago, IL, USA) and anti-mt-ValRS (15776-1-AP, ProteinTech group, Chicago, IL, USA); mouse monoclonal antibody anti-mitochondria extract clone MTC (UCS Diagnostic, Morlupo, Italy). Primary antibodies were visualized using secondary FITC-and Cy3-conjugated antibodies (Jackson Laboratories, Bar Harbor, ME, USA). The cells were examined and imaged with an Olympus IX50 Fluorescence microscope.

### Cell proliferation assay

To test growth capability, transformed cells were maintained in glucose medium for 24 h and then harvested and seeded at a constant number of cells (30 × 10^4^) in 60-mm dishes in glucose, galactose or GPM. Cell viability was measured by the Trypan blue dye exclusion assay. Cells were harvested after 24/48 h with 0.25% trypsin and 0.2% EDTA, washed, suspended in PBS in the presence of Trypan blue solution (Sigma) at a 1:1 ratio, and counted using a haemocytometer. For each time point, the number of viable cells in galactose was expressed as a percentage of the number of cells in glucose.

### Rate of apoptosis by flow cytometer

To test the rate of apoptosis transformed cells were maintained in glucose medium for 24 h and then harvested and seeded at 0.5–1 × 10^6^ in glucose, galactose or GPM for 24 h. Cells were washed and harvested in Binding Buffer 1X (BD Biosciences, Franklin Lakes, NJ, USA) by scraping to minimize potentially high annexin V background levels in adherent cells. Transfected cells were stained with APC-conjugated annexin V (BD Biosciences) and analysed on a FACS-Calibur flow cytometer (BD Biosciences) (Barbarulo *et al*, 2011).

### Respirometry assay

Maximum respiration rate (MRR) of m.4300A>G and m.4277T>C cybrids was measured with a XF96 Extracellular Flux Analyzer (Seahorse Bioscience, Billerica, MA, USA).

After transfection control and mutant m.4300A>G and m.4277T>C cybrids were maintained in glucose medium for 24 h then harvested and seeded in 8 wells of a XF 96-well cell culture microplate (Seahorse Bioscience) at a density of 10–15 × 10^3^ cells/well in 200 μl of medium. After incubation for 12 h at 37°C in 5% CO_2_ atmosphere, cells were either maintained in glucose medium or switched to galactose medium for 12 h. The growth medium was replaced with 180 μl of bicarbonate-free DMEM pre-warmed at 37°C for 30 min before starting the assay procedure. Maximum respiration rate (MRR) was assayed as previously described (Invernizzi *et al*, 2012). Data were expressed as pmol of O_2_ per minute and normalized by cell number measured by the CyQUANT Cell proliferation kit (Invitrogen™, Life Technologies), which is based on a fluorochrome binding to nucleic acids. Fluorescence was measured in a microplate luminometer with excitation wavelength at 485 ± 10 nm and emission detection wavelength at 530 ± 12.5 nm.

The rate of oxygen consumption of cybrids bearing the m.3243A>G mutation was evaluated with Clark type oxygen electrode (Hansatech Instruments, Norfolk, UK). After transfection control and mutant cybrids were maintained in glucose medium for 48 h, then OCR was measured in intact cells (5 × 10^6^) in 1.85 ml DMEM lacking glucose supplemented with 5% dialysed foetal bovine serum at 37°C, as previously described (Carelli *et al*, 2002).

### *In vitro* import

^35^S-methionine labeled peptides were generated from MTS-Cterm and Cterm cDNAs cloned into pcDNA3.2 using a TnT Quick Coupled Transcription-/Translation system™ (Promega, Fitchburg, WI, USA). The empty vector was also used as negative control for non-specific bands. Labelled proteins were incubated with rat liver mitochondria purified by differential centrifugation (Reyes *et al*, 2011) for 60  min at 37°C (Petruzzella *et al*, 1998). Products were resolved on 18% Tris-glycine NuPAGE SDS–PAGE gels, where one third to one sixth of the TnT amount used in each reaction was also loaded as control. After the run, the gels were dried and exposed to phosphorscreens for 5 days and scanned using a Typhoon™ phosphorimager (GE Healthcare, Life Sciences, Little Chalfont, UK). Additions were 300 μg/ml trypsin, to digest proteins outside mitochondria; FCCP (carbonylcyanide-p-trifluoromethoxyphenylhydrazone) to dissipate the mitochondrial membrane potential; 0.1% Triton X-100 to lyse the mitochondria. Mitochondrial binding was calculated as the proportion of the labeled peptide (input TnT, first lane of each panel) able to be pulled down or imported into either control or uncoupled mitochondria (second and fifth lane of each panel, respectively). Import efficiency was measured as the proportion of the labeled mitochondrial-interacting peptide (second and fifth lane of each panel) that was resistant to trypsin digestion in either control or uncoupled mitochondria (third or sixth lane of each panel, respectively).

### High resolution Northern

To determine the steady-state levels of mt-tRNA^Leu(UUR)^ WT, RNI64 and transiently trasformed cybrids were maintained in glucose medium for 12 h and then collected for RNA extraction. Total cytosolic RNA was obtained using Trizol reagent (Life Technologies, Paisley, UK). RNA (1 μg) was electrophoresed through 13% denaturing polyacrylamide gel, electroblotted onto Hybond N+ membrane (GE Healthcare Life Sciences, Little Chalfont, UK) in 0.25 × TBE and immobilized by ultraviolet cross-linking. Hybridization with radiolabelled probes was performed as previously described (Taylor *et al*, 2003).

### Surface Plasmon Resonance (SPR) experiments

Purified 5′-biotinylated human mt-tRNA^Leu(UUR)^ (bi-tRNALeu) and 5′-biotinylated mt-tRNA^Ile^ (bi-tRNAIle) were synthesized by Thermo Scientific (Illkirch, FR).

The interaction of bi-tRNA^Leu^ or bi-tRNA^Ile^ with GST-Cterm was studied with a BIACORE X system (Biacore AB, Uppsala, Sweden). Two streptavidin-coated dextran sensor chips (SD, Biacore AB) were chemically activated by a 35 μl injection of 10 mM NaOH, at a flow rate of 10 μl/min. Bi-tRNA^Leu^ and bi-tRNA^Ile^ were immobilized via interaction with the streptavidin surface of each chip. This procedure ensures directional immobilization of the bi-tRNAs via their 5′-termini. As a control, the sensor chips were treated as described above in the absence of bi-tRNAs. The interaction of immobilized bi-tRNA ligands with the GST-Cterm analyte was detected through mass concentration-dependent changes in the refractive index on the sensor chip surface, expressed as resonance units (RU). The increase in RU relative to baseline indicates complex formation. The plateau region represents the steady-state phase of the interaction, whereas the decrease in RU indicates dissociation of the GST-Cterm from the immobilized ligand after injection of buffer. A response change of 1,000 RU typically corresponds to a change in the protein concentration on the sensor chip of 1 ng/mm^2^.

The experiments were carried out at 298 K in degassed 20 mM HEPES at pH 7.4, 0.15 M NaCl, and 0.005% surfactant P-20 (HBS-P buffer). Measurements were performed at a flow rate of 30 μl/min with an immobilization level of about 500 RU of bi-tRNA^Leu^ in the first sensor chip, and about 550 RU of bi-tRNA^Ile^ in the second sensor chip. Values of the plateau signal at steady-state (Req) were calculated from kinetic evaluation of the sensorgrams using the BIA evaluation 4.1 software (Biacore-GE Healthcare, Life Sciences, Little Chalfont, UK). A Scatchard analysis of the dependence of Req on the concentration of analyte was also performed to assess the equilibrium constant of the interaction between bi-tRNAs and Cterm. Control experiments were carried out using GST as analyte.

### Bioinformatics analysis

Model building was performed with the program Phyre (http://www.sbg.bio.ic.ac.uk/phyre2/html/page.cgi?id=index). The three-dimensional structure of *E. coli* LeuRS available from the PDB (URL: http://www.rcsb.org; Berman *et al*, 2000) (PDB ID: 4ari, Resolution: 2.08 Å) was used as template.

Structure visualization and analysis were performed using PyMol (http://www.pymol.org/).

### Statistical analysis

All data are expressed as mean ± s.e.m. Data were analysed by standard ANOVA procedures followed by multiple pair-wise comparison adjusted with Bonferroni corrections. Significance was considered at *P* < 0.05. Numerical estimates were obtained with the GraphPad InStat 3 version (GraphPad Inc., San Diego, CA, USA).
