# Supplementary material for: The isolated carboxy-terminal domain of human mitochondrial leucyl-tRNA synthetase rescues the pathological phenotype of mitochondrial tRNA mutations in human cells
Source: EMBO Mol Med. 2014 Jan 10;6(2):169–82. doi: 10.1002/emmm.201303198 (PMC3927953; doi:10.1002/emmm.201303198)
Supplement: Supplementary file 1 [file emmm0006-0169-sd1.pdf]

EMBO Molecular Medicine

The isolated carboxy-terminal domain of human mitochondrial leucyl-tRNA synthetase rescues the pathological phenotype of mitochondrial tRNA mutations in human cells

Elena Perli, Carla Giordano, Annalinda Pisano, Arianna Montanari, Antonio F Campese, Aurelio Reyes, Daniele Ghezzi, Alessia Nasca, Helen A Tuppen, Maurizia Orlandi, Patrizio Di Micco, Elena Poser, Robert W Taylor, Gianni Colotti, Silvia Francisci, Veronica Morea, Laura Frontali, Massimo Zeviani, Giulia díAmati

Corresponding author: Giulia d'Amati, Sapienza University

Review timeline:

Submission date:	19 June 2013
Editorial Decision:	19 July 2013
Revision received:	16 September 2013
Accepted:	17 October 2013

Transaction Report:

(Note: With the exception of the correction of typographical or spelling errors that could be a source of ambiguity, letters and reports are not edited. The original formatting of letters and referee reports may not be reflected in this compilation.)

Editor: Roberto Buccione

1st Editorial Decision 19 July 2013

Thank you for the submission of your manuscript to EMBO Molecular Medicine. We have now received comments from the three Reviewers whom we asked to evaluate your manuscript.

Please note that we are experiencing a slight delay for one remaining evaluation for the accompanying manuscript EMM-2013-03202 "Human mitochondrial leucyl tRNA synthetase can suppress non-cognate pathogenic mt-tRNA mutations" by Hornig-Don et al. We have nevertheless decided to move forward with the present manuscript in the interest of time.

You will see that while all three Reviewers are generally supportive of your work and underline its considerable potential interest, they do seem to converge on the notion that there is insufficient mechanistic insight. I do agree that increasing the depth of mechanistic analysis would render your work more compelling and impactful.

Considered the above, we would be pleased to consider a suitably revised version that addressed the numerous technical and interpretational issues raised by Reviewer 1 and that in general furthered our mechanistic understanding of the findings, for instance concerning the hypotheis that the C-terminal fragment of LeuRS stabilizes mutant tRNA, as noted by Reviewers 2 and 3.

Please note that it is EMBO Molecular Medicine policy to allow a single round of revision only and that, therefore, acceptance or rejection of the manuscript will depend on the completeness of your

responses included in the next, final version of the manuscript.

I look forward to receiving your revised manuscript as soon as possible.

***** Reviewer's comments *****

Referee #1 (Remarks):

The authors here follow up on their recently-published findings that overexpression of mitochondrial tRNA synthetases (RS's) can ameliorate function in cells containing a mutation in both the cognate, and noncognate, mitochondrial tRNA. In this manuscript, they begin to address the mechanism by which such rescue might occur. First, they confirm these results with other full-length RS's, but more importantly, they show that only the C-terminus (~70 aa) of both the cognate and at least one non-cognate RS can do the job, whereas the remaining N-terminal region cannot. This is an important - and in the case of the non-cognate rescue, surprising - finding that has both basic and translational ramifications. However, given the potential impact of the work, I am surprised that a number of experimental issues were not addressed.

(1) The authors show that non-cognate C-term LeuRS can rescue MTT-Ile, but there was no "comparison control" showing that the cognate C-term IleRS itself can do the same. Please show this.

(2) I presume that the authors believe that the C-term goes to the inner membrane or matrix. Rather than appealing to human vs yeast models of the C-term to support the mitochondrial localization of the C-term, please express the C-term and show where it goes by submitochondrial fractionation, either "cold" (e.g. with appropriate antibodies, or with a C-terminal-epitope tag) or "hot" (e.g. with 35S-Met-labeled C-term).

(3) The surface plasmon resonance experiments are nice, but again, only explored binding of the cognate LeuRS C-term to tRNA-Leu. Where are the analogous non-cognate assays of LeuRS C-term to tRNA-Ile (or of IleRS C-term to tRNA-Leu, for that matter)? In a similar vein, if binding of the C-term to tRNA (whether cognate or non-cognate) is really happening, the binding should be able to be competed away (e.g. Ile C-term by Leu C-term, and vice versa).

(4) In the methods, I did not delve into the constructs in detail, so I may have missed it, but you need to explain better (or show with a figure) what the 67-aa C-term construct looks like (actually, aa 835-903 of human LARS2 would be 69 aa, not 67; please reconcile). Since aa 835-903 starts with a Gln (i.e. 835-QPEV...LVQD-903), where is the initiator Met? If from the vector, are there any other amino acids prior to aa-835 that could have targeted the C-term to mitos? Please clarify this.

(5) Besides qRT-PCR, please show the expression levels of the various full-length and C-term RS proteins by Western blot of total and mitochondrial fractions (if good antibodies are available). This is not a trivial matter, as the authors' previous paper (Perli et al., 2012) implied that there might be a quantitative (and inverse) relationship between the amount of RS and the degree of impairment in patient cells/tissues. This, in turn, implies some type of stoichiometric relationship between "free" and "RS-bound" forms of the tRNA.

(6) Formally, the rescue of viability could be non-mitochondrial in origin. Nowhere in the paper is there any analysis of mitochondrial function before and after treatment. For example, oxygen consumption, respiratory chain enzymology, ATP production, synthesis of mitochondrial polypeptides, enzyme histochemistry. While the authors need not do all of these assays, please pick one or two to confirm that the C-term truly rescues mitochondrial function.

(7) Minor issues: In Fig. S1, there is only one WT line shown compared to the two mutant (4277 and 4300) lines, implying that the WT line is a "generic" WT that is not isogenic with either of the two mutants (consistent with the inability to obtain a paired cybrid for a homoplasmic mutation). How representative is this WT line (i.e. did you assay more than one WT line [e.g. the 3 lines listed in Table S1?] to assess the range of viabilities that they have)? Also in Table S1, did you double-check that the MELAS mutant line was indeed homoplasmic (the line appears to be at least 21 years old)?

In Fig. 1 (and elsewhere), please identify the Y-axis as "...galactose/glucose."

Referee #2 (Remarks):

The authors extend their previous studies and those done in yeast to demonstrate that human mitochondrial leucyl- and valyl-tRNA synthetases can rescue the effect of non-cognate mutant tRNA in human cybrids. Furthermore they show that leucyl-tRNA synthetase and its C-terminal fragment can partially rescue the MELAS mutant cybrids. These findings are interesting considering the lack of treatment for mitochondrial tRNA disorders and the importance of MELAS especially. Although perhaps unexpected, the authors clearly demonstrate that the C-terminal fragment localizes to mitochondria.

Comments:
1.The study lacks mechanistical investigation and only demonstrates rescue by cell viability and bioenergetics. The authors speculate that the C-terminal fragment stabilizes the mutant tRNA. This definitely needs to be addressed for all mutants, especially MELAS.
2. Considering the therapeutic potential of the C-terminal fragment or its derivatives, does it bind to the cytoplasmic tRNAs and is there a danger of interfering with the cytoplasmic translation?

Referee #3 (Remarks):

The paper by D'Amati and coworkers is an interesting extension of previous studies on a path towards rescuing pathogenic mitochondrial tRNA mutations that occur in the human population. Previous work showed that overexpression of the cognate tRNA synthetase suppresses the mutant phenotype. In yeast, earlier work showed that a specific region of the synthetase (leucyl-tRNA synthetase (LeuRS) in case) was "necessary and sufficient". In this extension of that work, the authors showed in human cells that the same specific piece of the synthetase is sufficient to achieve rescue. This piece lacks catalytic activity and is derived from the C-terminal domain. All of this work is done using osteosarcoma-derived cybrid cells, in which the cybrid mitochondria harbor the homoplasmic pathogenic tRNA mutation. The authors go on to show that this C-terminal piece binds relatively well to a specific isoacceptor of wild-type tRNALeu in vitro. Based on these observations they speculate that the C-terminal piece of LeuRS stabilizes the structure of the mutant tRNA, and it is that stabilization rescues the phenotype.

The work is presented in a clear and straightforward way and, as far as it goes, appears to be well done. It is demonstrative and not definitive, mainly because they do not 'drill down' to validate their concluding hypothesis. For example, other structure-specific tRNA binding proteins, such as Trpb111 and Arc1p could have been tested, to see if they too rescue the phenotype. In addition, relatively direct experiments could be implemented to test the enhanced stability of the tRNA in the context of the bound protein. And finally, the authors could have used different methods to see if the pathogenic mutant tRNA(s) bound in vitro to the C-terminal piece of LeuRS and the structure-specific tRNA binding mutations.

The work has implications for human therapeutic interventions for these mitochondrial tRNA-based orphan diseases. For that reason, and because an extension of prior work into human systems is important, the paper is worthy of consideration for publication. But it would be a much more satisfying paper if the authors had gone a bit deeper.

© EMBO

1st Revision - authors' response 16 September 2013

Reviewer 1.

Comment

The authors show that non-cognate C-term LeuRS can rescue MTT-Ile, but there was no "comparison control" showing that the cognate C-term IleRS itself can do the same. Please show this.

Response

We thank the Reviewer for his/her observations. We actually omitted the reciprocal test (i.e. the effect of IleRS carboxy-terminal region overexpression in tRNA$^{Leu(UUR)}$ mutants) because preliminary data obtained in yeast showed that, while the overexpression of whole mt IleRS (ISM1) can rescue the defects due to mutations in non cognate tRNA^{Leu}, no rescue was observed with the carboxy-terminal of ISM1. In particular, the carboxy-terminal domain of ISM1 is incapable of rescuing the defective phenotype associated with mutations T32C in the cognate tRNA^{Ile} and C25T in the non-cognate tRNA^{Leu} (these mutations have been chosen because they are equivalent to human pathological mutations m.4290T>C in *MTTI*, m.3256C>T in *MTTL1*). We are pleased to provide a Figure illustrating our results (Supporting Information Figure 9).

Accordingly, our analysis of mt LeuRS and IleRS sequences and structures indicates that the properties of LeuRS Cterm cannot be directly transferred to the carboxy-terminal domain of mt IleRS Cterm. In particular: i) there is no detectable sequence similarity between mt-LeuRS and mt-IleRS from human or yeast in the carboxy-terminal region (Supporting Information Figures 10A and 10B); ii) since the amino acid sequences of human mt LeuRS and IleRS are highly similar to those of bacterial homologues whose three-dimensional structure is known (> 35% sequence identity), their structure and tRNA binding mode are predicted to be conserved. Analysis of the three-dimensional structures of LeuRS from *E. coli* and *T. thermophilus* and IleRS from *S. aureus* reveals relevant differences in both topology and tRNA binding mode (Supporting Information Figure 10C). In fact, while the carboxy-terminal region of LeuRS binds the elbow region of tRNA^{Leu} (see also Discussion of this and of the accompanying paper), the carboxy-terminal region of IleRS binds the anticodon region of tRNA^{Ile}.

In light of the above, we believe that the main determinants of the rescuing ability of human IleRS reside in regions different from the C-term. We have added a comment, summarising the above, at the end of the revised Discussion section. The identification of these determinants, and of the rescuing mechanism of IleRS and other aaRSs is certainly worth investigating and part of our future plans. However, this remains a challenging task, and is beyond the scopes of the present manuscript.

Comment

I presume that the authors believe that the C-term goes to the inner membrane or matrix. Rather than appealing to human vs yeast models of the C-term to support the mitochondrial localization of the C-term, please express the C-term and show where it goes by submitochondrial fractionation, either "cold" (e.g. with appropriate antibodies, or with a C-terminal-epitope tag) or "hot" (e.g. with 35S-Met-labeled C-term).

Response

We wholeheartedly agree with the Reviewer on this point. In the revised version of the manuscript we now provide quantitative data relative to the mitochondrial import of radiolabeled *in vitro*-translated Cterm, either with or without the mitochondrial targeting sequence. As illustrated in the revised Results section (Revised Figure 4 B) the Cterm domain was imported in a membrane potential-dependent manner with an efficiency of ~30%. The presence of the MTS improved the efficiency of mitochondrial import up to ~54%. Interestingly, the relative increase in mitochondrial import efficiency does not result in higher rescuing activity of the MTS-Cterm. In fact, overexpression of the Cterm, either with or without the MTS produced comparable effects on viability, apoptotic rate, and oxygen consumption of the 100% mutant m.3243A>G cybrids (Revised Figure 5).

<u>*Comment*</u>

The surface plasmon resonance experiments are nice, but again, only explored binding of the cognate LeuRS C-term to tRNA-Leu. Where are the analogous non-cognate assays of LeuRS C-term to tRNA-Ile (or of IleRS C-term to tRNA-Leu, for that matter)? In a similar vein, if binding of the C-term to tRNA (whether cognate or non-cognate) is really happening, the binding should be able to be competed away (e.g. Ile C-term by Leu C-term, and vice versa).

<u>Response</u>

According to the Reviewer's suggestion, in the revised version of the manuscript we now provide the results of surface plasmon resonance experiments showing that LeuRS C-term is able to directly interact also with biotinylated mt-tRNA^{Ile} , although with a lower (about 4 fold) affinity (Revised Figure 6 B, C). We omitted to explore the interaction of IleRS carboxy-terminal region with cognate and non-cognate mt-tRNAs for the reasons explained above (see the response to the first comment).

<u>*Comment*</u>

In the methods, I did not delve into the constructs in detail, so I may have missed it, but you need to explain better (or show with a figure) what the 67-aa C-term construct looks like (actually, aa 835-903 of human LARS2 would be 69 aa, not 67; please reconcile). Since aa 835-903 starts with a Gln (i.e. 835-QPEV...LVQD-903), where is the initiator Met? If from the vector, are there any other amino acids prior to aa-835 that could have targeted the C-term to mitos? Please clarify this.

<u>Response</u>

We apologise for the lack of clarity in the description of these constructs. We have now increased the detail to indicate how the construct was generated with an initiator methionine preceding the final 67 amino acids of LARS2. There is only the addition of a single methionine prior to the 67 amino acids of the C-terminus with no other extraneous sequence that could influence targeting to mitochondria.

<u>*Comment*</u>

Besides qRT-PCR, please show the expression levels of the various full-length and C-term RS proteins by Western blot of total and mitochondrial fractions (if good antibodies are available). This is not a trivial matter, as the authors' previous paper (Perli et al., 2012) implied that there might be a quantitative (and inverse) relationship between the amount of RS and the degree of impairment in patient cells/tissues. This, in turn, implies some type of stoichiometric relationship between "free" and "RS-bound" forms of the tRNA.

<u>Response</u>

We thank the Reviewer for his/her observation. Indeed, in our previous paper (Perli et al., 2012) we have shown *in vivo* a direct relationship between the steady state levels of mutated tRNA^{ile} , the endogenous levels of the cognate IleRS and the clinical/biochemical phenotype of the homoplasmic m.4277T>C mutation in *MTTI* gene. In fact, both IleRS mRNA/protein levels and mutated tRNA^{ile} steady-state levels were higher in fibroblasts and skeletal muscle from the unaffected mother as compared with the affected child. However, in the same paper, the direct relationship between phenotype and mutated tRNA steady-state levels was much less evident when the mutation was transferred in *trans*mitochondrial cybrids. This is in line with previous literature dealing with the same cell model.

Nevertheless, in the present manuscript, we looked at the steady-state levels of mutated mt-tRNA$^{Leu(UUR)}$ on 3243-cybrids, showing a ~50% reduction as compared with wild type (Supporting Information Figure 6). Transient overexpression with either the whole LeuRS enzyme, the Cterm domain or the MTS-Cterm did not result in a detectable increase of the mutated mt-tRNALeu(UUR) steady-state levels despite a clear functional rescue. For this reason we did not evaluate the expression levels of full-length and C-term LeuRS proteins.

We have added a section in the revised Discussion to comment on the results of the Northern experiments.

© EMBO

Comment

Formally, the rescue of viability could be non-mitochondrial in origin. Nowhere in the paper is there any analysis of mitochondrial function before and after treatment. For example, oxygen consumption, respiratory chain enzymology, ATP production, synthesis of mitochondrial polypeptides, enzyme histochemistry. While the authors need not do all of these assays, please pick one or two to confirm that the C-term truly rescues mitochondrial function.

Response

We apologize with the Reviewer for not clearly highlighting in the original manuscript our experiments investigating mitochondrial function (e.g. oxygen consumption) in the cybrids before and after rescue.

We hope these are now more clearly illustrated in the Results section both for the *MTTI* and *MTTL1* cybrids. Accordingly, we have also changed the legend titles for Figures 2 and 3.

The rate of oxygen consumption of cybrids bearing the m.3243A>G mutation was evaluated with Clark-type oxygen electrode. To detect the more subtle dysfunction of *MTTI* cybrids we used microscale oxygraphy. We hope that these experiments are now cleary illustrated and explained in the revised Results section.

Comment

Minor issues: In Fig. S1, there is only one WT line shown compared to the two mutant (4277 and 4300) lines, implying that the WT line is a "generic" WT that is not isogenic with either of the two mutants (consistent with the inability to obtain a paired cybrid for a homoplasmic mutation). How representative is this WT line (i.e. did you assay more than one WT line [e.g. the 3 lines listed in Table S1?] to assess the range of viabilities that they have)? Also in Table S1, did you double-check that the MELAS mutant line was indeed homoplasmic (the line appears to be at least 21 years old)? In Fig. 1 (and elsewhere), please identify the Y-axis as "...galactose/glucose."

Response

We thank the Reviewer for these observations and comments. The three control WT lines used listed in Table S1 belong to different haplotypes (J1, K1, and H5 respectively) and show similar growth capability and oxygen consumption rates, that are clearly different from those of mutant cybrids (see also Perli et al, 2012). The number of WT lines assessed for each experiment is provided in the figure legends.

We did double-check that the m.3242A>G mutant line was homoplasmic by pyrosequencing and have now added these results to the Supporting material (Supporting Information Figure 3) altering the text accordingly.

We identified the Y-axis as "...galactose/glucose" in all the figures, as suggested.

Reviewer 2

Comment

The study lacks mechanistical investigation and only demonstrates rescue by cell viability and bioenergetics. The authors speculate that the C-terminal fragment stabilizes the mutant tRNA. This definitely needs to be addressed for all mutants, especially MELAS.

Response

We agree with the Reviewer's observation. We performed high resolution northern hybridization to assess the steady-state levels of mutated mt-tRNA$^{Leu(UUR)}$ in MELAS (m.3243A>G) cybrids. Unexpectedly, transient overexpression with either the whole LeuRS enzyme, the Cterm domain or the MTS-Cterm did not result in a detectable increase of the mutated mt-tRNA$^{Leu(UUR)}$ steady-state levels as compared to the controls, despite a clear functional rescue. We added a section in the

revised Discussion to comment on this result and provide some explanation for these observations. We are painfully aware of the importance of a deeper mechanistic investigation into the interaction between the Cterm and the mutated mt-tRNAs, and this work is continuing in our laboratories. This is a challenging task, and one we feel goes beyond the scopes of the present manuscript which itself presents new and substantial data.

Comment

Considering the therapeutic potential of the C-terminal fragment or its derivatives, does it bind to the cytoplasmic tRNAs and is there a danger of interfering with the cytoplasmic translation?

Response

Although a specific effect on cytoplasmic translation has not been measured, so far the Cterm has not shown adverse side-effects on our cellular systems.

Additionally, mitochondrial tRNAs are characterized by the presence of unusual structural features and absence of nucleotides that are generally conserved in cytoplasmic tRNAs (see for example Helm M et al. (2000): "Search for characteristic structural features of mammalian mitochondrial tRNAs" RNA, 6: 1356–1379]. In particular, the variable (V) loop of human cytoplasmic $tRNA^{Leu(UUR)}$, which is one of the tRNA regions in contact with the carboxy-terminal domain of LeuRS in known three-dimensional structures (PDB IDs: 2bte and 4arc, see also text and answer to point 1 raised by reviewer 1), is much longer than that of the mitochondrial counterpart (14 vs. 5 nucleotides, respectively) [see sequences from the tRNA and mitochondrial tRNA databases at: http://mttrna.bioinf.uni-leipzig.de/]. Such relevant sequence and structural differences are likely to deeply affect Cterm binding.

Nevertheless, we agree that, to fully assess the therapeutic potential of the Cterm or its derivatives, their effect on physiological cellular activities, including cytoplasmic translation, will have to be tested. A systematic investigation of the ability of the Cterm and/or its derivatives to bind the other mitochondrial tRNAs, as well as their cytoplasmic counterparts, is also among our programs, with the aim to identify additional tRNA mutations that would benefit from the Cterm rescuing activity, in addition to potentially unwanted binding activities.

Finally, as mentioned in the discussion, the implementation of efficient mitochondrial targeting systems will be essential to minimize potential unwanted cytoplasmic effects.

Reviewer 3

Comment

For example, other structure-specific tRNA binding proteins, such as Trpb111 and Arc1p could have been tested, to see if they too rescue the phenotype. In addition, relatively direct experiments could be implemented to test the enhanced stability of the tRNA in the context of the bound protein. And finally, the authors could have used different methods to see if the pathogenic mutant tRNA(s) bound in vitro to the C-terminal piece of LeuRS and the structure-specific tRNA binding mutations.

Response

We thank the Reviewer for his/her interesting comments. The search for further tRNA binding proteins able to rescue the mitochondrial defects is certainly one of our goals. In the present paper we focused on mt aaRS based both on previous results in yeast human equivalent mutations and on the capability of these molecules to selectively target mitochondria.

Regarding the experiments to test the enhanced stability of the tRNA in the context of the bound protein we performed high resolution northern hybridization to assess the steady-state levels of mutated mt-$tRNA^{Leu(UUR)}$ on MELAS cybrids. Unexpectedly, transient overexpression with either the whole LeuRS enzyme, the Cterm domain or the MTS-Cterm did not result in a detectable increase of the mutated mt-$tRNA^{Leu(UUR)}$ steady-state levels as compared to the controls, despite a

clear functional rescue. We added a section in the revised Discussion to comment on this result. We appreciate that further work is requie to determine the specific molecular mechanisms relating to Cterm interaction with mutated mt-tRNAs and this work is continuing.

Acceptance 17 October 2013

Please find enclosed the final report on your manuscript. We are pleased to inform you that your manuscript is accepted for publication and is being sent to our publisher to be included in the next available issue of EMBO Molecular Medicine.

Congratulations on your interesting work.

***** Reviewer's comments *****

Is now suitable for publication.
