# Supplementary material for: The isolated carboxy-terminal domain of human mitochondrial leucyl-tRNA synthetase rescues the pathological phenotype of mitochondrial tRNA mutations in human cells
Source: EMBO Mol Med. 2014 Jan 10;6(2):169–82. doi: 10.1002/emmm.201303198 (PMC3927953; doi:10.1002/emmm.201303198)
Supplement: Supplementary file 2 [file emmm0006-0169-sd2.pdf]

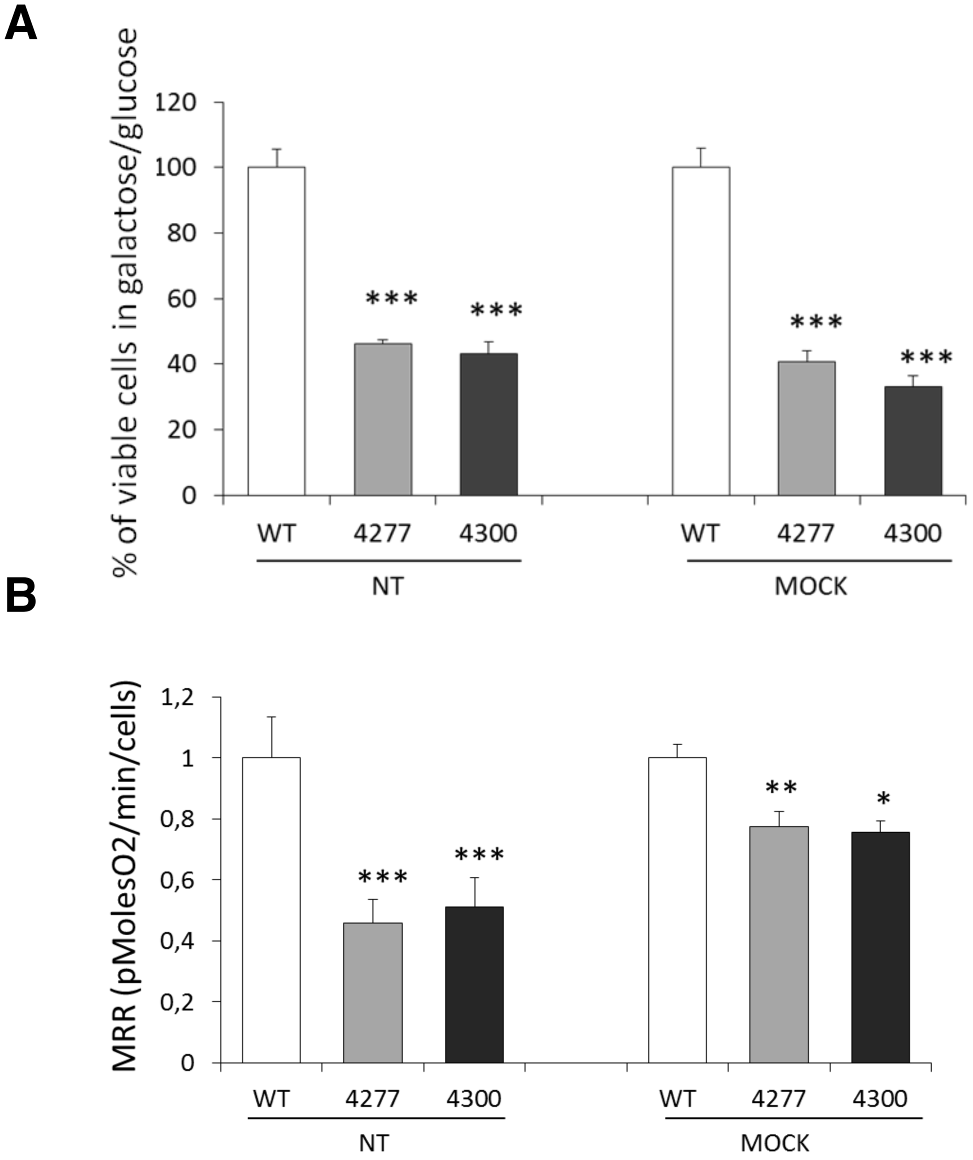

Supporting Information Figure 1. Pathological phenotype of m.4277T>C and m. 4300A>G *MTTI* mutant cybrids.

A. Viability of non transfected (NT) and mock transfected cells in galactose medium. Cells were mantained in glucose medium for 24 hours and then harvested and plated (30×10^4) in either glucose or galactose medium. The number of viable cells in galactose medium was evaluated after 48 hours and normalized for the number of viable cells in glucose at the same time point. After normalization, viability of mutant cybrids was expressed as a percentage of viability of wild type (WT) cybrids. Results are the mean of triplicate experiments on two 4277, two 4300 and two WT cell lines.

B. Maximal respiration rate (MRR) measured in non transfected (NT) and mock transfected cells. MRR values are expressed as ratio of MRR in galactose medium and MRR in glucose medium. After normalization, viability of mutant cybrids was expressed as a percentage of MRR of WT cybrids. Results are the mean of 6 replicates on one cell line for each mutation and for the WT.

* $p<0.05$, ** $p< 0.01$, *** $p< 0.001$ for mutant cybrids versus WT cybrids.
