# Supplementary material for: The isolated carboxy-terminal domain of human mitochondrial leucyl-tRNA synthetase rescues the pathological phenotype of mitochondrial tRNA mutations in human cells
Source: EMBO Mol Med. 2014 Jan 10;6(2):169–82. doi: 10.1002/emmm.201303198 (PMC3927953; doi:10.1002/emmm.201303198)
Supplement: Supplementary file 3 [file emmm0006-0169-sd3.pdf]

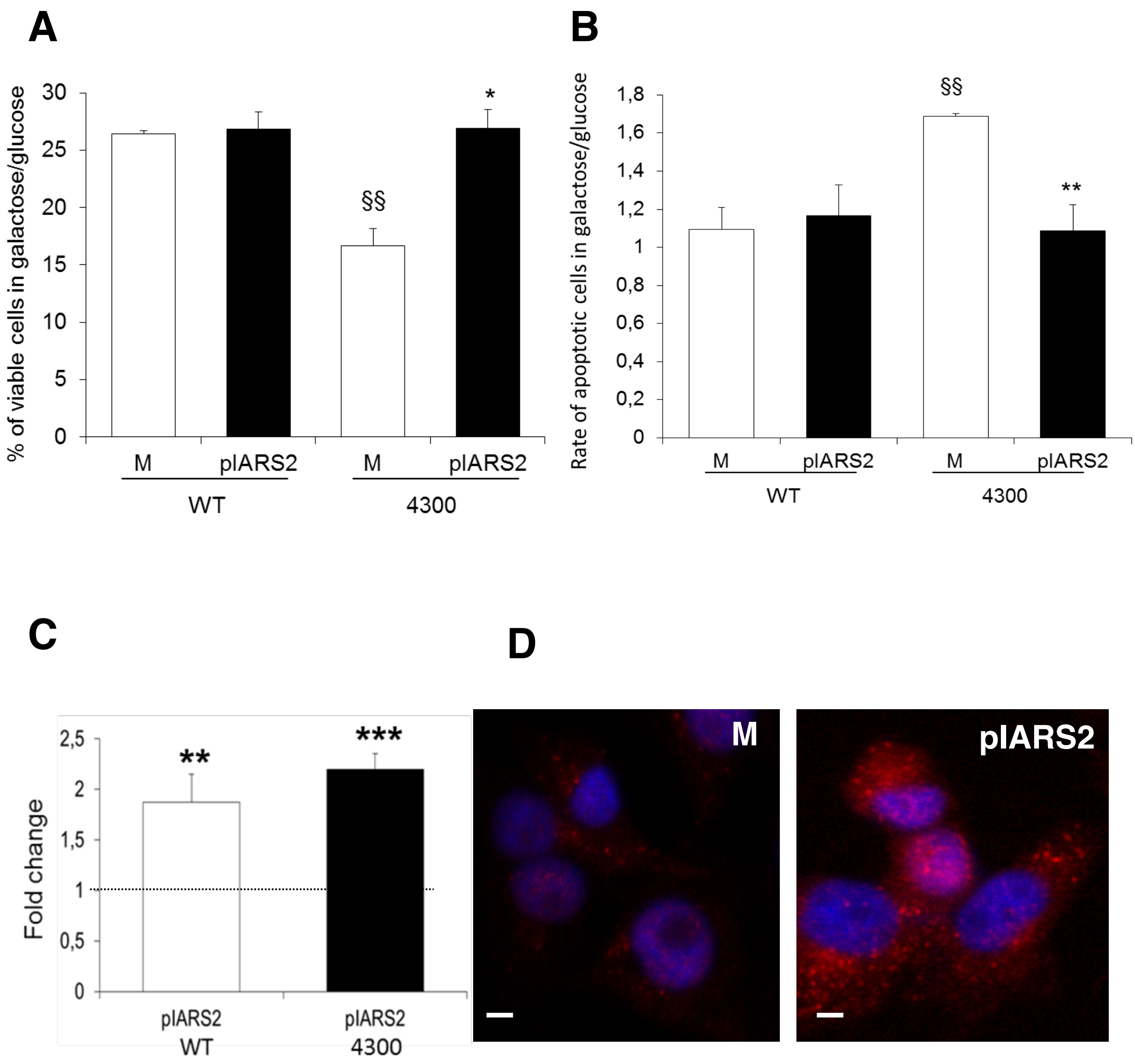

Supporting Information Figure 2. Phenotype correction of m.4300A>G *MTTI* cybrids by overexpressing the cognate human mt-IleRS.
A. Viability of mock and IleRS transformants (M and pIARS2) evaluated after 48 hours incubation in galactose medium. The number of viable cells in galactose is normalized to the number of viable cells in glucose at the same time point. **B.** Apoptotic cell death of M and pIARS2 evaluated after 24 hours incubation in galactose medium and expressed as a ratio between the percentage of apoptotic cells in galactose and in glucose medium. **C.** Relative expression levels of *IARS2* gene with respect to *HPRT1* gene. Gene expression levels are normalized to the gene expression level of respective mock. **D.** Immunofluorescence of M and pIARS2 with a specific anti mt-IleRS antibody. Nuclei are stained with DAPI. (Scale bar: 15 μm).
Results are the mean of triplicate experiments on one 4300 and one WT cell line.
* $p<0.05$, **$p< 0.01$, ***$p< 0.001$ for transformant cybrids versus mock cybrids.
§§ $p<0.001$for mutant versus WT mock cells (ANOVA test)
