# Supplementary material for: The isolated carboxy-terminal domain of human mitochondrial leucyl-tRNA synthetase rescues the pathological phenotype of mitochondrial tRNA mutations in human cells
Source: EMBO Mol Med. 2014 Jan 10;6(2):169–82. doi: 10.1002/emmm.201303198 (PMC3927953; doi:10.1002/emmm.201303198)
Supplement: Supplementary file 4 [file emmm0006-0169-sd4.pdf]

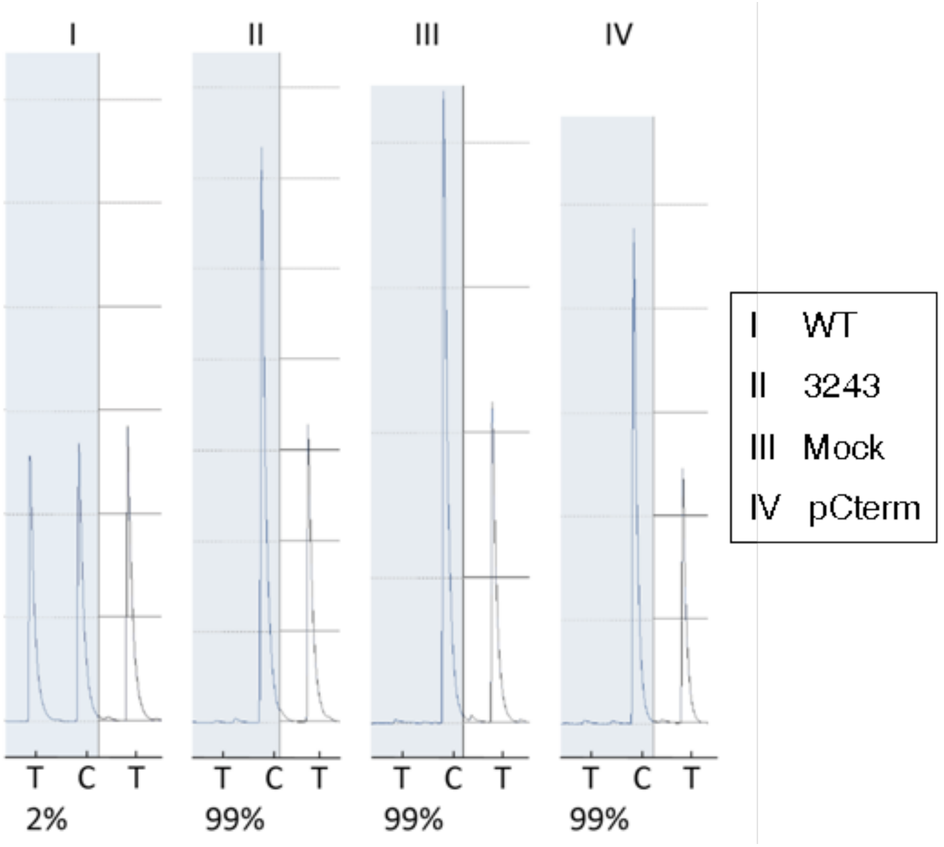

Supporting Information Figure 3. Pyrograms illustrating m.3243A>G heteroplasmy levels in different transmitochondrial cells. (I) WT cybrids, (II) cybrids bearing the m. 3243A>G mutation, (III) Mock transfected m.3243A>G cybrids and (IV) pCterm transfected m.3243A>G cybrids. Reverse H strand sequence is shown. Relevant peak heights for calculating mutation load are highlighted in blue and percentage levels of m. 3243A>G are indicated. The assay is sensitive to 1% mutation load, confirming the absence of the m.3243A>G mutation in WT cybrids and homoplasmic levels of the m. 3243A>G mutation in all m.3243A>G cybrid cell lines.
