# Supplementary material for: The isolated carboxy-terminal domain of human mitochondrial leucyl-tRNA synthetase rescues the pathological phenotype of mitochondrial tRNA mutations in human cells
Source: EMBO Mol Med. 2014 Jan 10;6(2):169–82. doi: 10.1002/emmm.201303198 (PMC3927953; doi:10.1002/emmm.201303198)
Supplement: Supplementary file 5 [file emmm0006-0169-sd5.pdf]

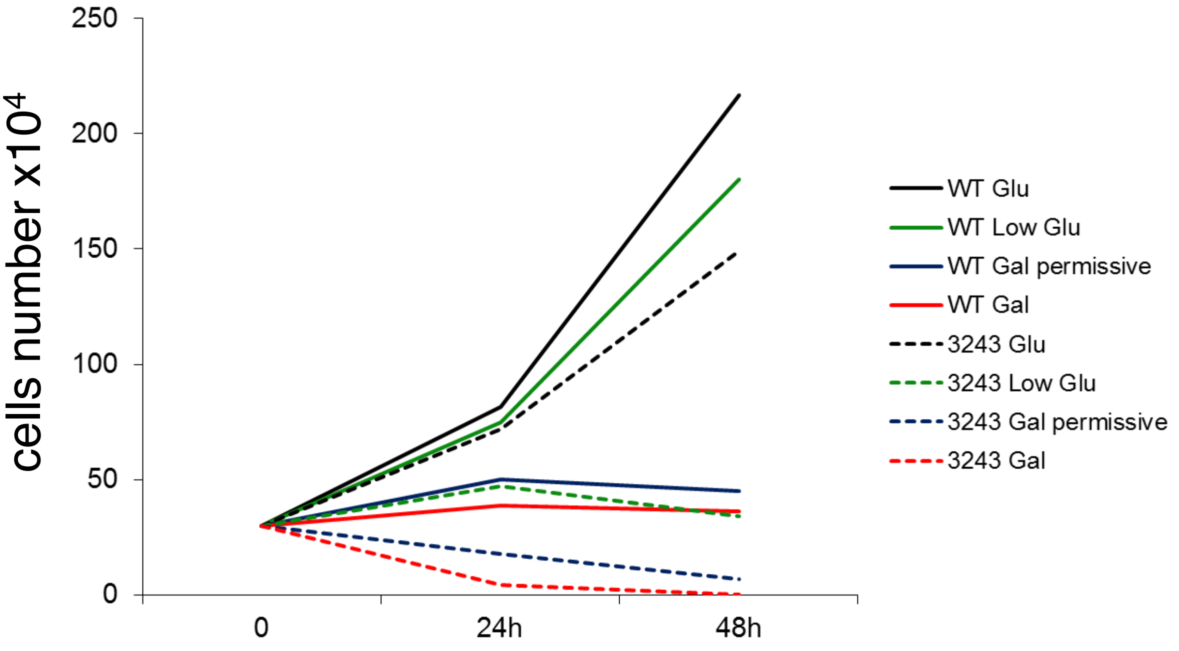

Supporting Information Figure 4. Growth rate of cybrids bearing the m. 3243A>G mutation in different culture conditions. Growth conditions: Glu (DMEM glucose [4,5g/L], FBS 10%); Low Glu (DMEM glucose [1g/L], FBS 10%); Gal permissive (DMEM free glucose, galactose supplemented [4,5g/L], FBS 10%); Gal (DMEM free glucose, galactose supplemented [4,5g/L], dialyzed FBS 10%).
