# Supplementary material for: The isolated carboxy-terminal domain of human mitochondrial leucyl-tRNA synthetase rescues the pathological phenotype of mitochondrial tRNA mutations in human cells
Source: EMBO Mol Med. 2014 Jan 10;6(2):169–82. doi: 10.1002/emmm.201303198 (PMC3927953; doi:10.1002/emmm.201303198)
Supplement: Supplementary file 6 [file emmm0006-0169-sd6.pdf]

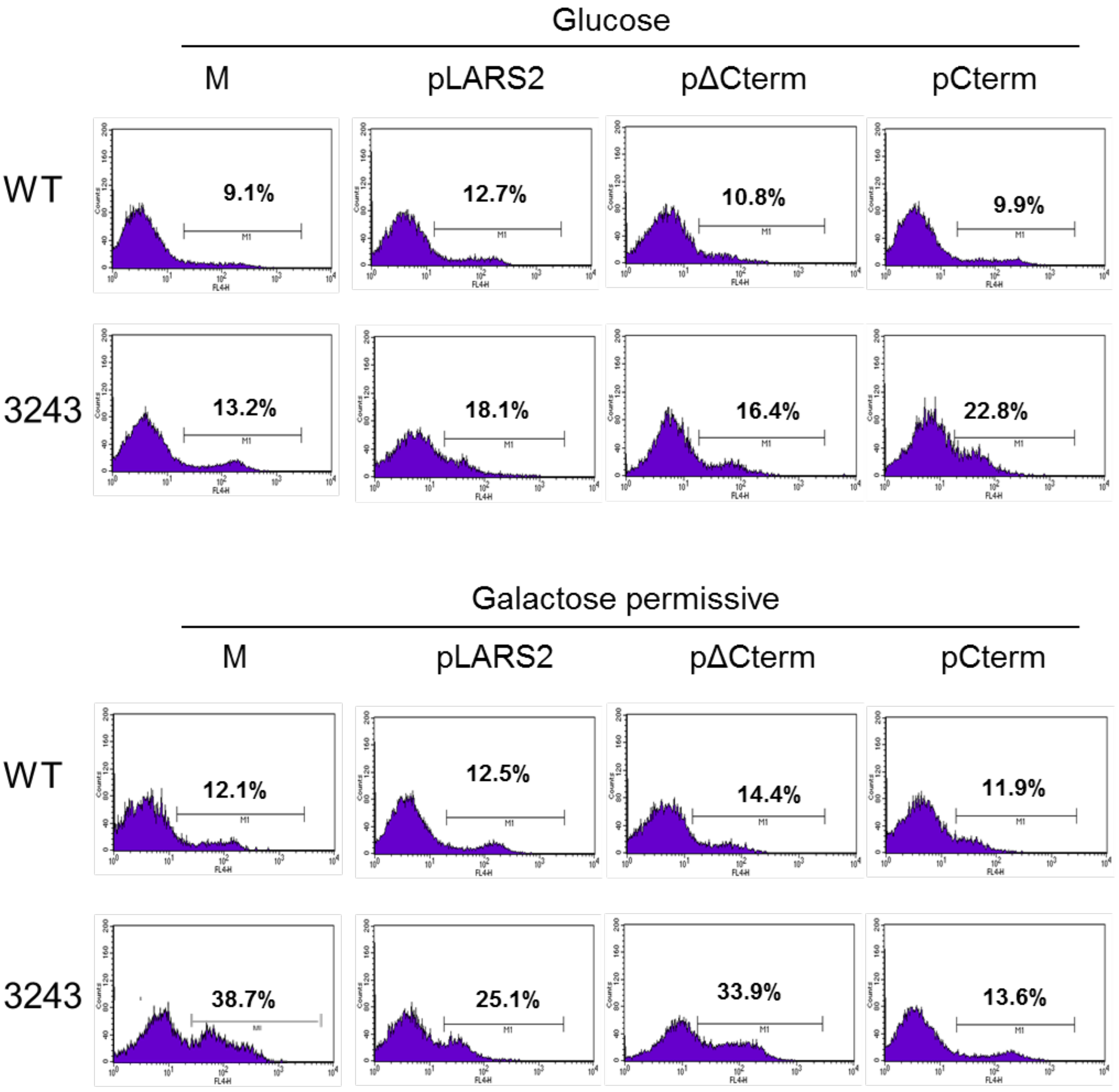

Supporting Information Figure 5. Representative plots of annexin V-APC staining of transformed cybrids by flow cytometry.
Cells were incubated in glucose or galactose permissive medium for 24 hours before staining. Inside each plot gray bars indicate the percentage of apoptotic cells in different conditions. Plots are representative of three repeated experiments.
