# Supplementary material for: The isolated carboxy-terminal domain of human mitochondrial leucyl-tRNA synthetase rescues the pathological phenotype of mitochondrial tRNA mutations in human cells
Source: EMBO Mol Med. 2014 Jan 10;6(2):169–82. doi: 10.1002/emmm.201303198 (PMC3927953; doi:10.1002/emmm.201303198)
Supplement: Supplementary file 7 [file emmm0006-0169-sd7.pdf]

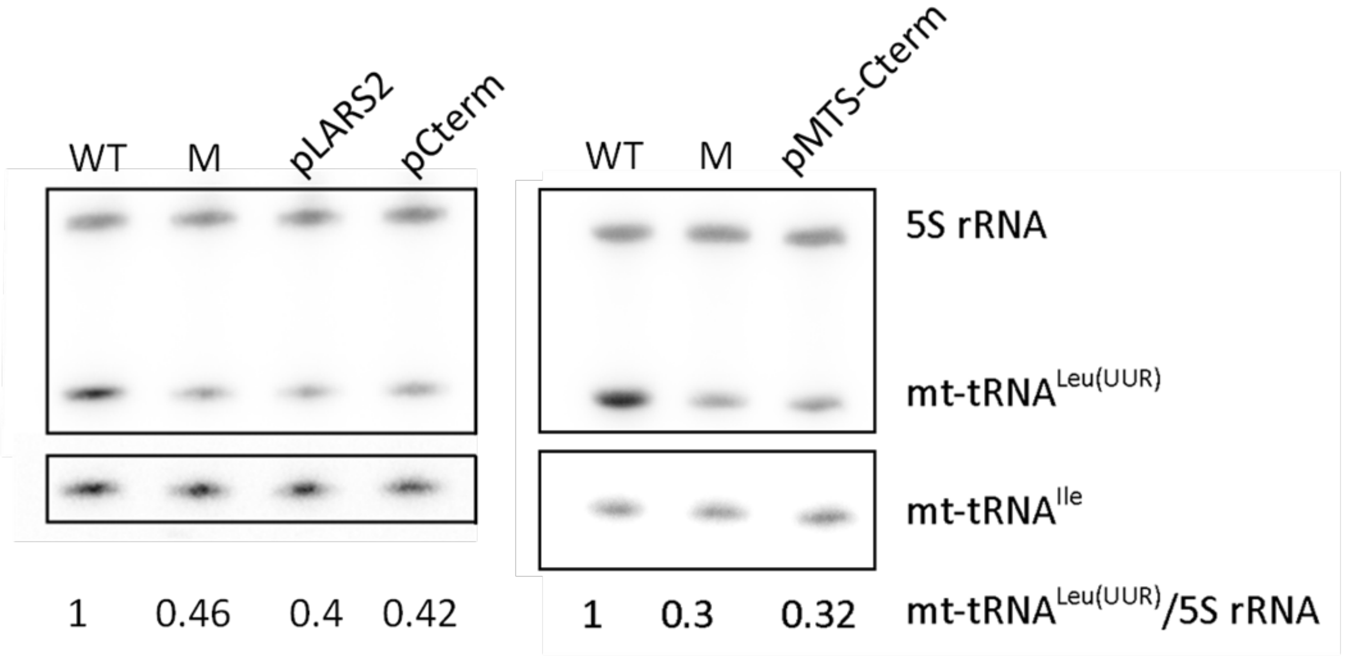

Supporting Information Figure 6. Steady state levels of mutant mt-tRNALeu$^{(UUR)}$ in transiently transfected cybrids. Mutant cybrids transfected with the whole LeuRS enzyme (pLARS2) the Cterm domain (pCterm) and the MTS-Cterm domain (pMTS-Cterm) were maintained in glucose medium for 12 hours before RNA extraction. RNA (1µg) was electrophoresed through 13% denaturing polyacrylamide gel and hybridized with radiolabelled probes for 5S-rRNA, mt-tRNA^{Ile} and mt-tRNALeu$^{(UUR)}$. Transfection with either of the constructs does not result in a detectable increase in mutated tRNA steady state levels, as compared to the mock (M).
