# Supplementary material for: The isolated carboxy-terminal domain of human mitochondrial leucyl-tRNA synthetase rescues the pathological phenotype of mitochondrial tRNA mutations in human cells
Source: EMBO Mol Med. 2014 Jan 10;6(2):169–82. doi: 10.1002/emmm.201303198 (PMC3927953; doi:10.1002/emmm.201303198)
Supplement: Supplementary file 8 [file emmm0006-0169-sd8.pdf]

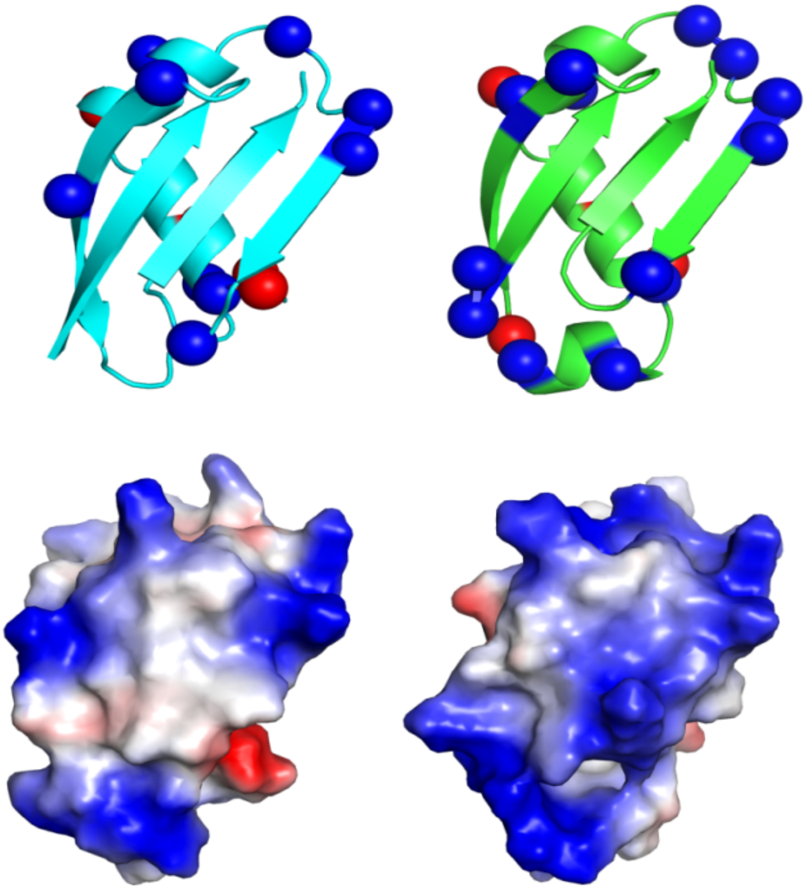

Supporting Information Figure 7. Three-dimensional model of human (left) and yeast (right) mt-LeuRS Cterm domains. Top: ribbon representation of the Cα atoms (cyan and green for human and yeast Cterm, respectively). Positively and negatively charged residues are shown as spheres and coloured blue and red, respectively. Bottom: qualitative electrostatic potential calculated by PyMol. Positively and negatively charged regions are coloured blue and red, respectively.
