# Supplementary material for: The isolated carboxy-terminal domain of human mitochondrial leucyl-tRNA synthetase rescues the pathological phenotype of mitochondrial tRNA mutations in human cells
Source: EMBO Mol Med. 2014 Jan 10;6(2):169–82. doi: 10.1002/emmm.201303198 (PMC3927953; doi:10.1002/emmm.201303198)
Supplement: Supplementary file 9 [file emmm0006-0169-sd9.pdf]

A

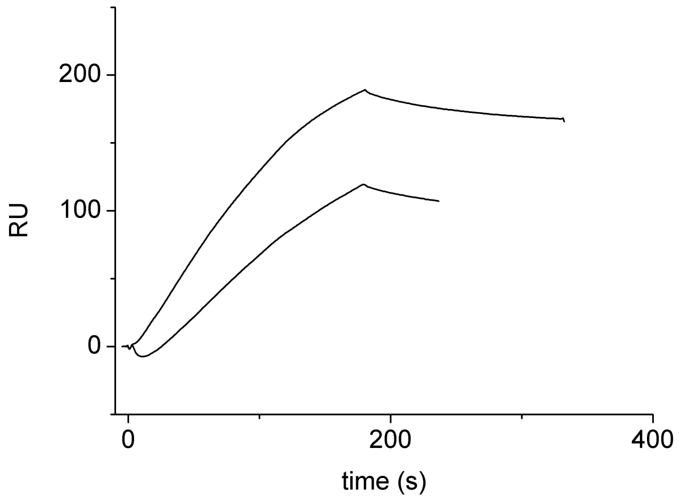

B

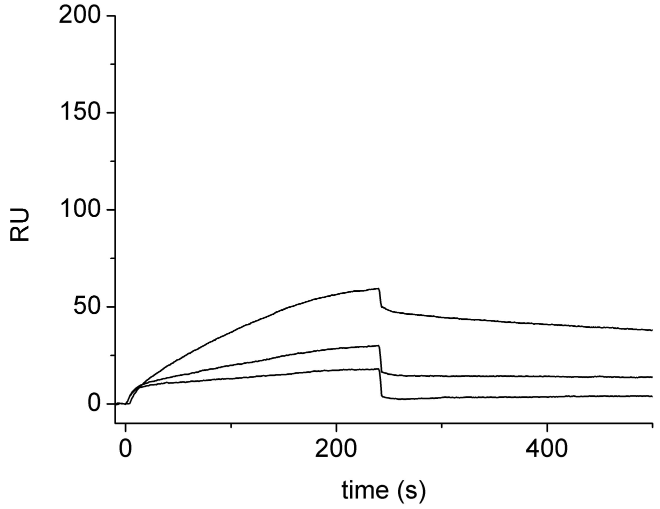

Supporting Information Figure 8. Surface Plasmon Resonance experiments measure the interaction of GST with 5'-biotinylated mt-tRNA$^{Leu(UUR)}$ and 5'-biotinylated mt-tRNA^{Ile}.

A. Control experiment, in which the 5'-biotinylated mt-tRNA$^{Leu(UUR)}$ was immobilized on a streptavidin-coated sensorchip, carried out in 20 mM HEPES, 150 mM NaCl, 0.005% P20 surfactant, pH 7.4, at 298 K. Sensorgrams were obtained using GST at 600 nM and 2.5 μM concentration as analyte. The signal is about 25% of that obtained using the same concentrations of GST-Cterm as analyte. Additionally, the association process is slower and the dissociation process is faster than in the sensorgrams in Figure 6, showing the interaction between GST-Cterm and 5'-biotinylated mt-tRNA$^{Leu(UUR)}$. **B.** Control experiment, in which the 5'-biotinylated mt-tRNA^{Ile} was immobilized on a streptavidin-coated sensorchip, carried out in 20 mM HEPES, 150 mM NaCl, 0.005% P20 surfactant, pH 7.4, at 298 K. Sensorgrams were obtained using GST at 0.6 μM, 1.2 μM and 2.5 μM concentration as analyte. The signal is about 15% of that obtained using the same concentrations of GST-Cterm as analyte.
