# Supplementary material for: The isolated carboxy-terminal domain of human mitochondrial leucyl-tRNA synthetase rescues the pathological phenotype of mitochondrial tRNA mutations in human cells
Source: EMBO Mol Med. 2014 Jan 10;6(2):169–82. doi: 10.1002/emmm.201303198 (PMC3927953; doi:10.1002/emmm.201303198)
Supplement: Supplementary file 10 [file emmm0006-0169-sd10.pdf]

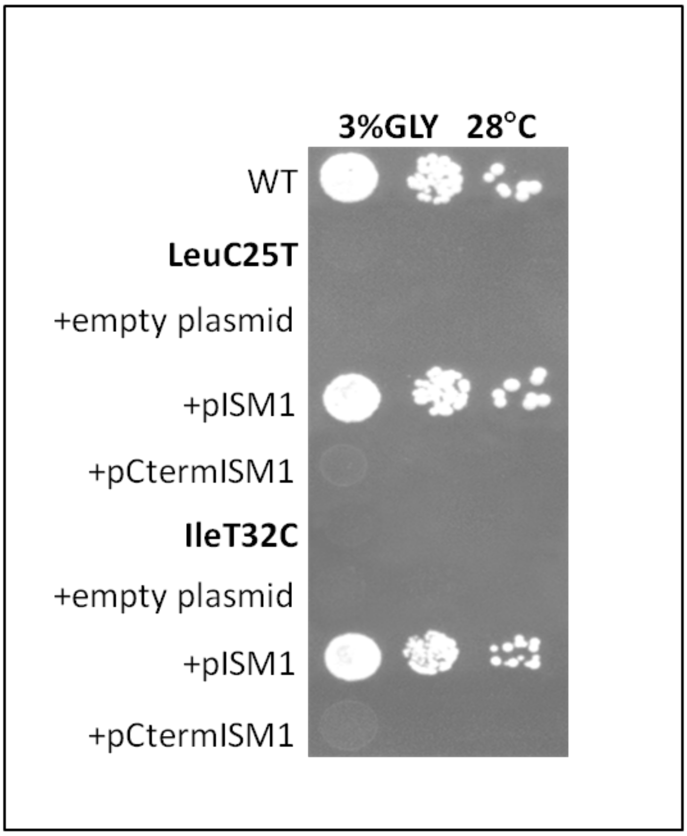

Supporting Information Figure 9. The carboxy-terminal region of IleRS is not able to rescue the defective growth phenotype of yeast mt tRNA mutants. Growth capability of yeast WT (MCC123) and mutants (LeuC25T and IleT32C) transformed or not with plasmids (empty or bearing either the full lenght *ISM1* gene coding for mt-IleRS, or the carboxy-terminal region). Serial dilutions were plated on glycerol containing media and depicted after five days of growth at 28°C. For yeast Materials and Methods see Montanari et al, 2011.
