# Supplementary material for: The isolated carboxy-terminal domain of human mitochondrial leucyl-tRNA synthetase rescues the pathological phenotype of mitochondrial tRNA mutations in human cells
Source: EMBO Mol Med. 2014 Jan 10;6(2):169–82. doi: 10.1002/emmm.201303198 (PMC3927953; doi:10.1002/emmm.201303198)
Supplement: Supplementary file 11 [file emmm0006-0169-sd11.pdf]

A. Human mt-LeuRS and mt-IleRS alignement

```
Sc-LeuRS ----------MLSRPSSRFLSTK-----------RGPGPA-----VKKLIAIGEK--WKQ  32
Sc-IleRS MKRSRLVPQHIFSIISKRYLAKHAYQKTLNLPKTKFPNRSNLEITLRELIPKSSQLVYKE  60
                     *  * * *                *           **        *

Sc-LeuRS KTTRGLPKQDTLNSGSK---------YILCQFP-YPSGALHIGHLRVYVISDSLNRFYKQ  82
Sc-IleRS QLRDFFEEFSKLNTTDEKLEFIKEKLFILHDGPPYANGELHLGHALNKILKDIINRYQLS 120
                    **              **   * *  * ** **       *  **

Sc-LeuRS KGYNVIHPMGWDAFGLPAENAAIE-------RSINPAI-------WTRDNIAKMKQQMQS 128
Sc-IleRS QGKYIFYKPGWDCHGLPIEIKALKDLSAQQIESISPLKIRSMALKHAQKAIKRQRETFQH 180
          *       ***  *** *  *          ** *              *       *

Sc-LeuRS MLANFDWDREITTCDPEYYKFTQWIFLKLFENGLAYRKEAEINWDPVDMTVLANEQVDAQ 188
Sc-IleRS FAILTDWETPYLTMDKDYEINQLNIFKEMYERGLIKRQNKPVYWGTETRTALAEGELE-Y 239
              **     * *  *      **    * ** *      *     * **

Sc-LeuRS GRSWRSGAIVEKKQLKQWFLGITKFAPKLKKHLNQLKDWPSNVKQMQKNWIGESVGAELV 248
Sc-IleRS NENHKSIAAYVKFPLEK------KSQMDLCKKLGITNNLPIYC----------------- 276
              * *   *  *        *    * * *      *

Sc-LeuRS FKVADPKFENLIVFTTRPETLFAVQYVALALD--HPIVQKYCEEMPDLKEFIQKSDQLPN 306
Sc-IleRS -----------LIWTSTPWTLLSNRAICFNQDFSYSLLRLNSELILVETGSIDKLGLTTN 325
                       *  * **          *          *        * *     *

Sc-LeuRS --DTKEGFQLPNIKAV---NPLTKEEV--PIFAAPYVVSSYGSAPSAVMGCPGHDNRDFE 359
Sc-IleRS SFETIKQFQGTHLNGLYYQNLLVDDKVGRPLLHGAHVTS--GTGTGLVHTAPGHGQDDYL 383
         *  **        * *   * *     * * *     *  ***   *
Sc-LeuRS F-----------------WQTNCPGEHIKTCIAPFFD--DASKVTEQERQRII------- 393
Sc-IleRS IGIQNGLEIYSPVDHQGRYQLNELPQSVRSIVRDEGDLTKGRQVLDAETAKIILCKLSDL 443
                            * *              *      *   *   **

Sc-LeuRS ----------DTVPFT--STDGVLTKECGEHSGVLTVVARKSIMGMLN-----SEGLSKS 436
Sc-IleRS NLLYKSHEYTHSYPYDWRSKKPVIIRATPQWFADLHDVKNLALESISRVKFCPKRGYSRL 503
                      *    *   *           *  *                 * *

Sc-LeuRS VVRYKIR-DWLISRQRYWGTPIPIIHCDNCGPVPVPESDL---PVKLPELEGLDT----K 488
Sc-IleRS SSFMKSRNEWCISRQRSWGIPILSFYKKSEPDSVLMNSEILAHAIEKIKQKGINAWFNDK 563
             * *  * ***** ** **               *             *       *

Sc-LeuRS GNPLSTI--DEFVNVACPSCGSPAKRETDTMDTFIDS--SWYYFRFLDPKNTSKPFDREI 544
Sc-IleRS DNDMKEWLPEKYHDVAHEYC-----RSQDTMDVWFDSGSSWSVIKDF--------YEKSL 610
          *            **   *     *  ****   **  **

Sc-LeuRS ASKNMPVDIYIGGVEHAILHLLYSRFIAKFLGSINAWSDPAGIFEPFKKLVTQGMV--QG 602
Sc-IleRS KLSKLPSPLYQVCLEGSDQHRGW--FQSSLLTKVASSNVPVA---PYEEVITHGFTLDEN 665
              *   *    *    *     *    *        *     *     * *

Sc-LeuRS KTYVDPDSGKFLKPDELTFVNDS-------PDG-NTVIIKSNGKVPVVSYEKMSKSKYNG 654
Sc-IleRS GLKMSKSVGNTISPEAIIRGDENLGLPALGVDGLRYLIAQSNFTTDIV------------ 713
                 *    *                 **    *  **     *

Sc-LeuRS ADPNECILRH-GPDATRAHILFQSPIADALNWDESKIVGIERWLQKVLHLTKNILSLEKD 713
Sc-IleRS AGPT--VMKHVGEALKKVRLTFRYLLSNLQKSQDFNLLPIEQ-LRRV------------- 757
         * *      * *         *                 **  *  *

Sc-LeuRS LAISKDYKTPTDLNDAEVKFHNDFQRFLKS---ITESFEVNLSLNTVISDYMK--LTNIL 768
Sc-IleRS -----DQYTLYKINELLETTREHYQKYNFSKVLITLQYHLNNELSAFYFDISKDILYSNQ 812
              *  *    *          *    *   **     *  *     *  *  *

Sc-LeuRS ESALKKGEVRNEMIVQNLQKLVTVIYPAVPSISEEAAEMI-------NSQMEWNQYR--W 819
Sc-IleRS ISSLARRQVQTTL-VHILNAYRAILAPILPVMVQEVWKYIPEGWLQGQEHIDINPMRGKW 871
          * *    *     *  *        *  *    *    *             *  *  *

Sc-LeuRS PEVERTTE--SKFKKFQIVV--------------NGRVKFMYTA----DKNFLKLGRDAV 859
Sc-IleRS PFLDSNTEIVTSFENFELKILKQFQEEFKRLSLEEGVTKTTHSHVTIFTKHHLPFSSDEL 931
         *     **    *  *                   *  *          *  *    *

Sc-LeuRS IETLMN------------------LPEG-RMYLMNKKIKKF----VMKFN--------- 886
Sc-IleRS CDILQSSAVDILQMDDNNNSLPTIELGSGINVQILVERSKRHNCPRCWKANSAEEDKLCD 991
            *                     *  *          *        **

Sc-LeuRS ----VISFLFHK   894
Sc-IleRS RCKEAVDHLM-S  1002
             *
```

B. Yeast mt-LeuRS and mt-IleRS alignement

```
Hs-LeuRS --MASVW----QRLGFYASLLKRQLNGGPDVI---KWERRVIPGCTRSIYSAT-------  44
Hs-IleRS MR----WGLRPRGPGAAALATARSLWGTPRLPCSPGWQGATKRLLVRSVSGASNHQPNSN  56
               *       *  *    * * * *       *         **   *

Hs-LeuRS -GKWTKEYTL-QTRKDVEKWWHQRIK-----EQASKISEA------DKSKPKFYVLSMFP  91
Hs-IleRS SGRYRDTVLLPQTSFPMKLLGRQQPDTELEIQQKCGFSELYSWQRERKVKTEFCLHDGPP 116
          *       * **         *         *    **        * *  *      *

Hs-LeuRS YPSGKLHMGHVRVYTISDTIARFQKMRGMQVINPMGWDAFGLP------------AEN-A 138
Hs-IleRS YANGDPHVGHALNKILKDIANRFHMMNGSKIHFVPGWDCHGLPIEIKVLSELGREAQNLS 176
         *  *  * **       *   ** * *       ***  ***            * *

Hs-LeuRS AVERNLHPQSWTQSNIKHMRKQLDRLGLCFSWDREITTCLPDYYKWTQYLFIKLYEAGLA 198
Hs-IleRS AMEIRKKARSFAKAAIEKQKSAFIRWGIMADWNNCYYTFDGKYEAKQLRTFYQMYDKGLV 236
         * *      *     *        * *    *     *    *       *   *  **

Hs-LeuRS YQKEALVNWDPVDQTVLANEQVD---EHGC------------SWRSGAKVEQKYLRQWFI 243
Hs-IleRS YRSYKPVFWSPSSRTALAEAELEYNPEHVSRSIYVKFPLLKPSPKLASLIDGSSPVSILV 296
         *     * * *   * **        **              *                  .

Hs-LeuRS KTTA-YAKAMQDALADLPEW-YGIKGMQAHWIGDC----------VGCHLDFTLKVHGQA 291
Hs-IleRS WTTQPWTIPANEAVCYMPESKYAV--VKCSKSGDLYVLAADKVASVASTLETTFETISTL 354
          **         *    **  *          **           *   *  *

Hs-LeuRS TGEKLTAYTATPEAI-------YGTSHVAISPSHRLLH---GHSSLKEALRMALVPGKDC 341
Hs-IleRS SGVDLENGTCSHPLIPDKASPLLPANHVTMAKGTGLVHTAPAHGMEDYGVASQHNLPMDC 414
          *  *   *     *           **       * *    *               **

Hs-LeuRS LTPVMAVNMLTQQEVPVVILAKADLEGSLDSKIGIPSTSSEDTILAQTLGLAYSEVIETL 401
Hs-IleRS LVDEDGV--FTDVAGPE-LQNKAVLEEGTDVVIKMLQTA-KNLLKEEKLVHSYPYDWRTK 470
         *     *   *    *     ** **   *  *    *          *   *     *

Hs-LeuRS PDGTERLSSSAEFTGMTR-QDAFLALTQKAR---GKRVGGDVTS-DKLKDWLISRQRYWG 456
Hs-IleRS KPVVIR-ASKQWFINITDIKTAAKELLKKVKFIPGSALNGMVEMMDRRPYWCISRQRVWG 529
              *  *   *   *    *   *  *     *    * *   *    * ***** **

Hs-LeuRS TPIPIVHCPVCGPTPVPLEDLPVTLPNIASFTGKGG-------PPLAMASEWVNCSCPRC 509
Hs-IleRS VPIPVFHHKTKDEYLINSQ----TTEHIVKLVEQHGSDIWWTLPPEQLLPKEV---LSEV 582
          ***  *                *   *       *       **      *

Hs-LeuRS KGAAKRE----TDTMDTFVDS--AWYYFRYTDPHNPHSPFNTAVADYWMPVDLYIGGKEH 563
Hs-IleRS GGPDALEYVPGQDILDIWFDSGTSWSYVL-------PGPDQRA--------DLYLEGKDQ 627
          *    *     *  *   **   * *           *   *        ***  **

Hs-LeuRS AVMHLFYARFFSHFCHDQKMVKHREPFHKLLAQGLIKGQTFRLPSGQYLQREEVDLTGSV 623
Hs-IleRS L------GGWFQSSLLTSVAARKRAPYKTVIVHGFTLGEK-------------------- 661
                   *            **        *   *

Hs-LeuRS PVHAKTKEKLEVTWEKMSKSKHNGVDPEEVVE---------QYGIDTIRLYILFAAPPEK 674
Hs-IleRS -------------GEKMSKSLGNVIHPDVVVNGGQDQSKEPPYGADVLRWWVA-----DS 703
                 ******  *   *  **        ** *  *
Hs-LeuRS DILWDVKTDALPGVL---RWQQRLWTLTTRFIEARASGKSPQP------------QLLSN 719
Hs-IleRS NVFTEVAIG--PSVLNAARDDISKLRNTLRFLLGNVADFNPETDSIPVNDMYVIDQYMLH 761
              *     * **   *        * **         *              *

Hs-LeuRS KEKAEARKLWE-YKN----SVISQVTTHFTEDFS--LNSAISQLMGLSNALSQASQSVIL 772
Hs-IleRS LLQDLANKITELYKQYDFGKVVRLLRTFYTRELSNFYFSIIKDRLYCEKENDPKRRSCQT 821
              * *  * **      *     *  *   *    * *               *

Hs-LeuRS HSPEFEDALCALMVMAAPLAPHVTSEIWAGLALVPRKLCAHYT-WDASVLLQAWPAVDPE 831
Hs-IleRS ALVEILDVIVRSF---APILPHLAEEVFQHIPYIKEPKSVFRTGWISTSSIWKKPGLE-- 876
            *  *         **  **   *                 * *         *

Hs-LeuRS FLQQPEVVQMAVLINNKACGKIP-----------------------VPQQVARDQDKVH 867
Hs-IleRS -----EAVESACAMRDSFLGSIPGKNAAEYKVITVIEPGLLFEIIEMLQSEETSSTSQLN 931
              * *  *        * **

Hs-LeuRS EFVLQSELGVRL--------------------LQGRSIKKSF------------------ 889
Hs-IleRS ELMMASESTLLAQEPREMTADVIELKGKFLINLEGGDIREESSYKVIVMPTTKEKCPRCW 991
         *    **                         * *  *

Hs-LeuRS ---------LSPRTALINFLVQD  903
Hs-IleRS KYTAESSDTLCPRCAEV--VSGK 1012
             * ** *
```

C

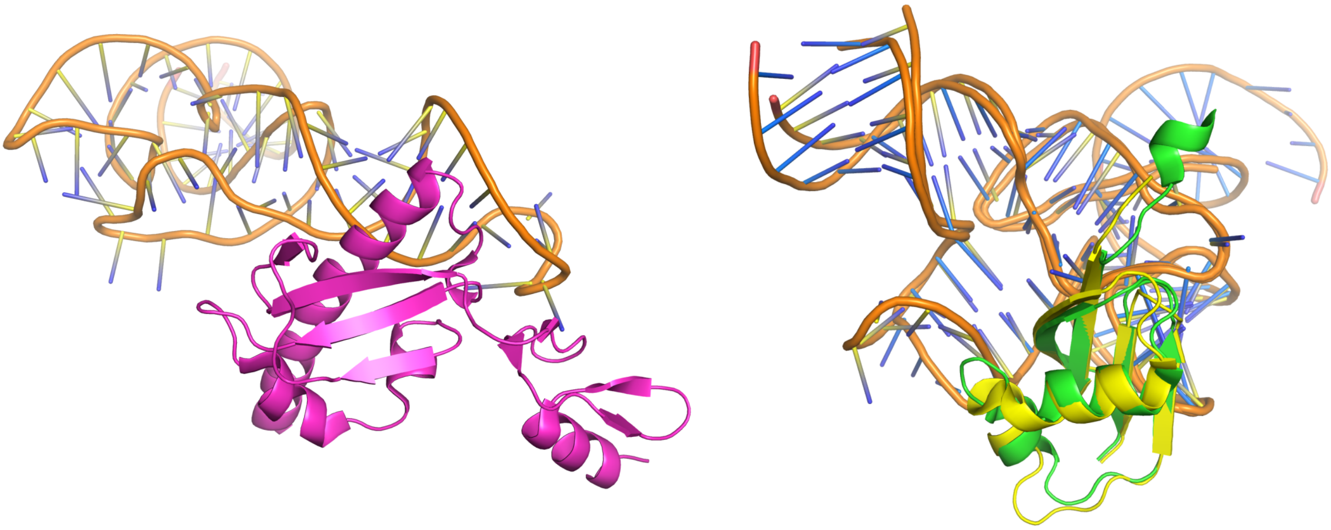

Supporting Information Figure 10. The carboxy-terminal region of IleRS differs from that of LeuRS in aminoacid sequence and three-dimensional structure.
A-B) Sequence alignment of mt-LeuRS and mt-IleRS from human (Hs-LeuRS, Hs-IleRS) and yeast (Sc-LeuRS, Sc-IleRS). Identical amino acids are indicated by asterisks. The Cterm domain of human (B) and yeast (C) mt-LeuRS is underlined. The percentages of sequence identity of the carboxy-terminal region, whole enzymes, and whole enzymes except for the carboxy-terminal regions are 9%, 15% and 15%, respectively, for human mt-LeuRS and mt-IleRS, and 12 %, 13 % and 13 %, respectively, for the yeast counterparts. The sequences of human mt-LeuRS (NP_056155.1) and mt-IleRS (NP_060530.3) **(A)** and of yeast mt-LeuRS (NP_013486.3) and mt-IleRS (NP_015285.1) **(B)** were downloaded from the NCBI (http://www.ncbi.nlm.nih.gov/). Pairwise sequence alignments were produced using the NW-align program (http://zhanglab.ccmb.med.umich.edu/NW-align), which is designed for global sequence alignments. Programs optimized for local sequence alignments (e.g., BLAST: http://blast.ncbi.nlm.nih.gov/Blast.cgi) do not include the carboxy-terminal regions in the alignment because their sequence similarity is too low.
C) Ribbon representation of the carboxy-terminal domains of *Staphylococcus aureus* IleRS (PDB ID: 1ffy), *Escherichia coli* and *Thermus thermophilus* LeuRS (PDB IDs: 4arc and 2bte, respectively). The carboxy-terminal domain of IleRS (left panel), coloured magenta, binds the anticodon region of the cognate tRNAIle. The superimposed carboxy-terminal domains of *E. coli* and *T. thermophilus* LeuRs (right panel), coloured yellow and green, respectively, are highly similar in structure and mode of binding of the elbow region of the cognate tRNALeu. The backbone and nucleotide bases of *S. aureus* tRNAIle bound to IleRS (top) and of both *E. coli* and *T. thermophilus* tRNALeu bound to LeuRSs (bottom) are shown as orange ribbon and magenta-and-blue sticks, respectively. The picture was generated by PyMol.
