# Supplementary material for: The isolated carboxy-terminal domain of human mitochondrial leucyl-tRNA synthetase rescues the pathological phenotype of mitochondrial tRNA mutations in human cells
Source: EMBO Mol Med. 2014 Jan 10;6(2):169–82. doi: 10.1002/emmm.201303198 (PMC3927953; doi:10.1002/emmm.201303198)
Supplement: Supplementary file 12 [file emmm0006-0169-sd12.pdf]

Supporting Information Table 1. Cybrid cell lines

Cell line	Mutation	Reference
HGA*	WT	*Ghelli et al. 2009*
HPC7*	WT	*Ghelli et al. 2009*
HP27*	WT	*Pello et al. 2008*
HPG8	m.4277T>C	*Perli et al. 2012*
HTM21	m.4277T>C	*Perli et al. 2012*
HRCAM10	m.4300A>G	*Perli et al. 2012*
HMDL7	m.4300A>G	*Perli et al. 2012*
RN164*	m.3243A>G	*King et al. 1992*

* Generous gift from Dr Monica Montopoli and Valerio Carelli
