# Supplementary material for: The isolated carboxy-terminal domain of human mitochondrial leucyl-tRNA synthetase rescues the pathological phenotype of mitochondrial tRNA mutations in human cells
Source: EMBO Mol Med. 2014 Jan 10;6(2):169–82. doi: 10.1002/emmm.201303198 (PMC3927953; doi:10.1002/emmm.201303198)
Supplement: Supplementary file 13 [file emmm0006-0169-sd13.pdf]

Supporting Information Table 2. Real-time PCR assays used to assess gene expression levels

Gene	Assay ID or primers and probe sequences
HPRT1	Hs99999909_m1
IARS2	Hs01058371_m1
VARS2	Hs00383681_m1
LARS2	Hs00209733_m1
C-term from *LARS2*	For 5’-GAGGTTGTCCAGATGGCA-3’<br>Rev 5’-GGCACAGGAATTTTGCCAC-3’<br>Probe 6-FAM-TCTGATCAACAATAAAG-MGB
