# Supplementary material for: The isolated carboxy-terminal domain of human mitochondrial leucyl-tRNA synthetase rescues the pathological phenotype of mitochondrial tRNA mutations in human cells
Source: EMBO Mol Med. 2014 Jan 10;6(2):169–82. doi: 10.1002/emmm.201303198 (PMC3927953; doi:10.1002/emmm.201303198)
Supplement: Supplementary file 14 [file emmm0006-0169-sd14.pdf]

Supporting Information References

Ghelli A, Porcelli AM, Zanna C, Vidoni S, Mattioli S, Barbieri A, Iommarini L, Pala M, Achilli A, Torroni A et al. (2009) The background of mitochondrial DNA haplogroup J increases the sensitivity of Leber's hereditary optic neuropathy cells to 2,5-hexanedione toxicity. PLoS One 4(11): e7922

King MP, Koga Y, Davidson M, Schon EA (1992) Defects in mitochondrial protein synthesis and respiratory chain activity segregate with the tRNA(Leu(UUR)) mutation associated with mitochondrial myopathy, encephalopathy, lactic acidosis, and strokelike episodes. Mol Cell Biol 12:480-490

Pello R, Martín MA, Carelli V, Nijtmans LG, Achilli A, Pala M, Torroni A, Gómez-Durán A, Ruiz-Pesini E, Martinuzzi A et al. (2008) Mitochondrial DNA background modulates the assembly kinetics of OXPHOS complexes in a cellular model of mitochondrial disease. Hum Mol Genet 17:4001-4011

Perli E, Giordano C, Tuppen HA, Montopoli M, Montanari A, Orlandi M, Pisano A, Catanzaro D, Caparrotta L, Musumeci B et al. (2012) Isoleucyl-tRNA synthetase levels modulate the penetrance of a homoplasmic m.4277T>C mitochondrial tRNAIle mutation causing hypertrophic cardiomyopathy. Hum Mol Genet 21:85-100

Montanari A, De Luca C, Di Micco P, Morea V, Frontali L, Francisci S (2011) Structural and functional role of bases 32 and 33 in the anticodon loop of yeast mitochondrial tRNAIle. RNA 17: 1983-1996
