# Supplementary material for: The isolated carboxy-terminal domain of human mitochondrial leucyl-tRNA synthetase rescues the pathological phenotype of mitochondrial tRNA mutations in human cells
Source: EMBO Mol Med. 2014 Jan 10;6(2):169–82. doi: 10.1002/emmm.201303198 (PMC3927953; doi:10.1002/emmm.201303198)
Supplement: Supplementary file 15 [file emmm0006-0169-sd15.pdf]

Supporting Information

Table of contents

Supporting Information Table 1 (page 2)

Supporting Information Figure 1 (page 3)

Supporting Information Figure 2 (page 4)

Supporting Information Figure 3 (page 5)

Supporting Information Figure 4 (page 6)

Supporting Information Figure 5 (page 7)

Supporting Information Figure 6 (page 8)

Supporting Information Figure 7 (page 9)

Supporting Information Figure 8 (page 10)

Supporting Information Figure 9 (page 11)

Supporting Information Figure10 (page 12-13)

Supporting Information Table 2 (page 14)

Supporting Information References (page 15)
